# Genome-wide analysis of CCCH zinc finger family in Arabidopsis and rice

**DOI:** 10.1186/1471-2164-9-44

**Published:** 2008-01-27

**Authors:** Dong Wang, Yinghui Guo, Changai Wu, Guodong Yang, Yingying Li, Chengchao Zheng

**Affiliations:** 1 State Key Laboratory of Crop Biology, College of Life Sciences, Shandong Agricultural University, Taian, Shandong 271018, P.R. China

## Abstract

**Background:**

Genes in the CCCH family encode zinc finger proteins containing the motif with three cysteines and one histidine residues. They have been known to play important roles in RNA processing as RNA-binding proteins in animals. To date, few plant CCCH proteins have been studied functionally.

**Results:**

In this study, a comprehensive computational analysis identified 68 and 67 CCCH family genes in Arabidopsis and rice, respectively. A complete overview of this gene family in Arabidopsis was presented, including the gene structures, phylogeny, protein motifs, and chromosome locations. In addition, a comparative analysis between these genes in Arabidopsis and rice was performed. These results revealed that the CCCH families in Arabidopsis and rice were divided into 11 and 8 subfamilies, respectively. The gene duplication contributed to the expansion of the CCCH gene family in Arabidopsis genome. Expression studies indicated that CCCH proteins exhibit a variety of expression patterns, suggesting diverse functions. Finally, evolutionary analysis showed that one subfamily is higher plant specific. The expression profile indicated that most members of this subfamily are regulated by abiotic or biotic stresses, suggesting that they could have an effective role in stress tolerance.

**Conclusion:**

Our comparative genomics analysis of CCCH genes and encoded proteins in two model plant species provides the first step towards the functional dissection of this emerging family of potential RNA-binding proteins.

## Background

Transcription factors are important regulators of cellular processes, and the complexity of living organisms necessitates a large number of transcription factors. The zinc finger motifs, which are classified based on the arrangement of the zinc-binding amino acids, are present in many transcription factors and play critical roles in interactions with other molecules [1,2]. A large amount of zinc-finger transcription factors are implicated in important biological processes and many of them often share common characteristic to form a family. So far, several zinc finger families have been found in plants, such as RING-finger, ERF, WRKY, DOF and LIM family [3-8]. However, most of them are identified as DNA-binding or protein-binding proteins, fewer function as RNA-binding proteins.

The CCCH zinc finger motif has been found in proteins from organisms ranging from man to yeast [9-18]. The CCCH proteins are a large family of zinc finger containing C3H-type motifs and many evidences proved that they may be RNA-binding proteins functioning in RNA processing [19-21]. In mouse, tristetraprolin, a protein containing two CCCH zinc fingers, binds directly to AU-rich elements within the 3'-untranslated region of target transcripts to facilitate mRNA degradation [22-25]. Zfp36l2, like its better-known relative TTP, is a mRNA-binding and destabilizing protein, functions in the physiological control of female fertility at the level of early embryonic development [26]. The PIE-1 is an essential regulator of *Caenorhabditis elegans *germ cell fate that segregates with the germ lineage by inhibition of transcription or activation of protein expression from maternal RNAs [27]. Compared to animals, only a few of plant CCCH proteins have been characterized functionally. In *Arabidopsis thaliana*, HUA1 has been proved to be a RNA-binding protein and likely participates in a new regulatory mechanism governing flower development [19]. *AtCPSF30 *encodes a small polypeptide which shares the probable ortholog of the 30-kD subunit of the mammalian cleavage and polyadenylation specificity factor, and it was shown to be nucleus-localized RNA-binding protein that binds calmodulin [20]. PEI1 is an embryo-specific CCCH zinc finger protein that plays an important role during Arabidopsis embryogenesis, functioning primarily in the apical domain of the embryo [28]. *FES1 *interacts genetically with *FRI *and *FRL1 *to promote the winter-annual habit of Arabidopsis and might be involved in the processing of mRNA [29]. In rice (*Oryza sativa*), OsDOS is a nuclear protein that delays leaf senescence by integrating developmental cues to the jasmonate (JA) pathway [30]. It was also proposed to play a role in posttranscriptional level by interacting with target RNA.

Given the potential for CCCH proteins to function diverse roles by associating with RNA and most of members containing CCCH motif remaining poorly understood, it was of considerable interest to us to characterize the plant CCCH gene family. The availability of the *Arabidopsis *and *Oryza sativa *genome sequences allows the genome-wide comparative analysis of gene families between monocot and eudicot plants [31-35].

Perl is one of the most widely used programming languages for managing and manipulating life-science information [36]. Perl has been extremely successful for connecting software applications together into sequence analysis pipelines, converting file formats, and extracting information from the output of analysis programs and other text files [37,38].

In this study, we wrote Perl program to search against the entire genome of two species and performed a genome-wide analysis of the CCCH gene family in *Oryza sativa *and *Arabidopsis*. A total of 68 CCCH genes from *Arabidopsis *and 67 from *Oryza sativa *were identified and most of them have not been reported previously. Phylogenetic analyses revealed that the CCCH families in *Arabidopsis *and *Oryza sativa *were divided into 11 and 8 subfamilies, respectively. Expression studies indicated that CCCH proteins exhibit a variety of expression patterns, suggesting diverse functions. Detailed analysis of the higher plant-specific subfamily IX of Arabidopsis revealed its involvement in response to various stresses. Our genomics analysis provides the framework for future studies to dissect the function of this emerging family of potential RNA-binding proteins.

## Results and discussion

### Identification of genes coding for CCCH zinc finger protein in Arabidopsis

**Figure 1:**
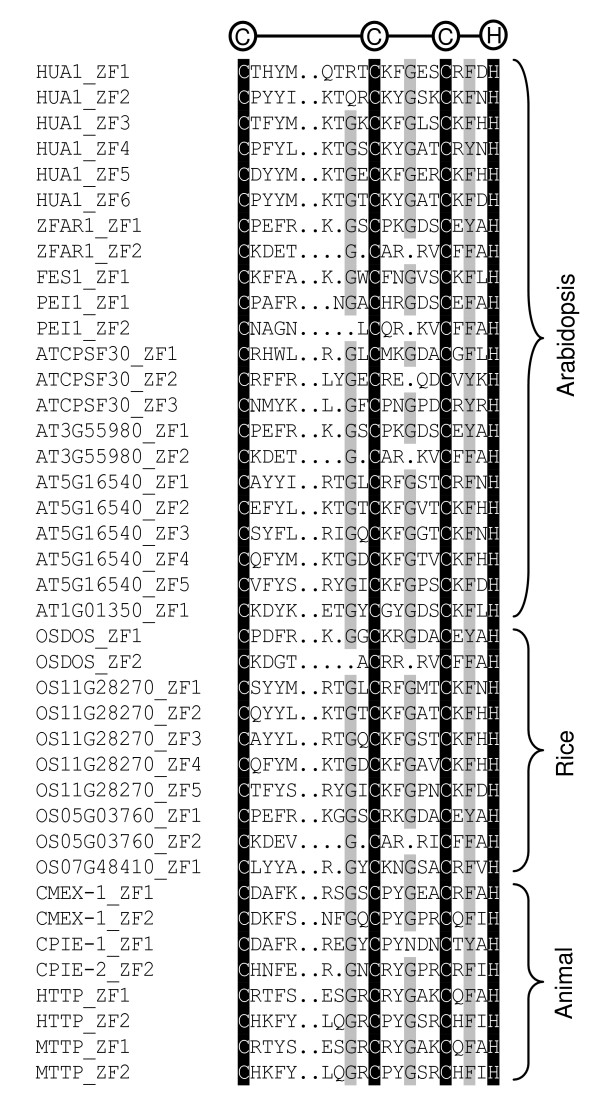
**Alignment of the CCCH zinc finger motifs from selected CCCH proteins**. Black and light grey shading indicate identical and conserved amino acid residues, respectively. The three cysteine and one histidine residues putatively responsible for the zinc-finger structure are indicated.

The previous research revealed that CCCH zinc finger protein contained 1–6 copies of C3H-type zinc-finger motifs characterized by three Cys and one His (Figure 1). Berg et al. defined that the CCCH family is a group of zinc-finger protein consisting of canonical C-X-C-X-C-X-H motif (C-X_6–14_-C-X_4–5_-C-X_3_-H) [21]. Based on the different amino acid spacing numbers between Cys and His in zinc finger motif, we divided the family into 18 groups (Table 1). The protein containing the motif within these groups was considered as a candidate member of CCCH family.

**Table 1:** The potential CCCH motifs.

**Group**		**Arabidopsis**		**Rice**	
		
		Predicted by Perl	After examining by Pfam/SMART	Predicted by Perl	After examining by Pfam/SMART
A	C-X_4_-C-X_5_-C-X_3_H^b^	3	2	6	0
B	C-X_5_-C-X_4_-C-X_3_H^b^	12	12	14	9
C	C-X_6_-C-X_4_-C-X_3_H	6	0	15	0
D	C-X_6_-C-X_5_-C-X_3_H	9	0	26	0
E	C-X_7_-C-X_4_-C-X_3_H	18	5	17	6
F	C-X_7_-C-X_5_-C-X_3_H	43	42	66	35
G	C-X_7_-C-X_6_-C-X_3_H^b^	5	1	11	1
H	C-X_8_-C-X_4_-C-X_3_H	10	2	19	1
I	C-X_8_-C-X_5_-C-X_3_H	46	44	56	36
J	C-X_8_-C-X_6_-C-X_3_H^b^	6	1	11	0
K	C-X_9_-C-X_4_-C-X_3_H	9	0	410^a^	0
L	C-X_9_-C-X_5_-C-X_3_H	11	4	14	6
M	C-X_10_-C-X_4_-C-X_3_H	9	0	24	0
N	C-X_10_-C-X_5_-C-X_3_H	13	1	9	3
O	C-X_11_-C-X_4_-C-X_3_H	10	0	12	0
P	C-X_11_-C-X_5_-C-X_3_H	7	1	8	1
Q	C-X_12_-C-X_4_-C-X_3_H	29	0	9	0
R	C-X_12_-C-X_5_-C-X_3_H	9	0	7	0
S	C-X_13_-C-X_4_-C-X_3_H	5	0	8	0
T	C-X_13_-C-X_5_-C-X_3_H	17	0	28	0
U	C-X_14_-C-X_4_-C-X_3_H	4	0	8	0
V	C-X_14_-C-X_5_-C-X_3_H	12	0	4	0
W	C-X_15_-C-X_5_-C-X_3_H^b^				1

^a ^The proteins containing the catalytic triad Cys-His-Asn domain.

^b ^The CCCH motifs that were not referred in the studies of Berg et al. [21].

**Figure 2:**
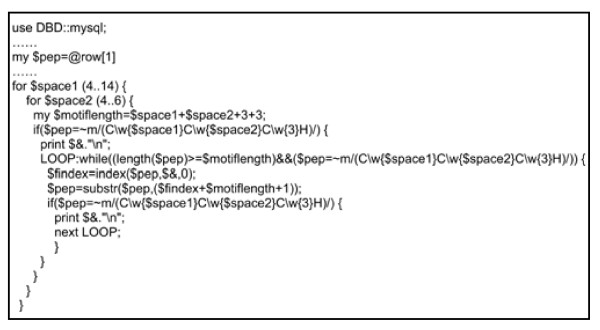
**The program for detecting the CCCH motifs by regular expression**. The code was used to detect the proteins containing CCCH motifs from local MySQL database. The whole program is available in additional file (see Additional file 5 and 7).

To uncover the entire family of genes coding for CCCH zinc finger protein in Arabidopsis, we analyzed the proteome data with a program written by Perl (Figure 2, see Additional file 1, 2, 3), in which a regular expression (C\w{6,14}C\w{4,5}C\w{3}H) was used to search for the CCCH motifs. Sequences that matched the criteria within 18 groups could be easily detected by the program and were considered as putative CCCH proteins.

In addition, to verify the reliability of our result, we performed multiple BLAST algorithms to search against the corresponding data set using the known CCCH proteins got from several database as our query (see Methods). Another program written by Perl was used to parse the results and exclude the redundant entries from the initial data set.

The Perl program could identify all the amino acid sequences included the ones which do not belong to CCCH family. For instance, the sequences "CIRLEAYEDCIEYFVCLNGH" in AT5G15440 could be detected as a C-X_8_-C-X_5_-C-X_3_-H motif. Our comparison analysis showed that the entries obtained from Perl program contained all the entries obtained from BLAST searches. Thus, the data got from Perl program were used for further analysis.

Subsequently, we performed SMART and Pfam analysis to find the putative CCCH protein sequences [39,40]. Seventy-nine proteins with typical CCCH motif encoded by 68 genes were detected from the original data, and five of them, AT1G19860, AT2G16470, AT2G33835, AT3G51120 and AT5G12440, contained a predicted CCCH motif with low confidence values. The proteins which were not detected by SMART and Pfam were eliminated from our study. For AT3G51180, which could not be detected by SMART and Pfam but has a sister gene in genome duplication region and contains a C-X_8_-C-X_5_-C-X_3_-H motif, was also considered as a member of CCCH zinc-finger family. In addition, we wrote another program to analyse the type of CCCH motifs based on the residue number between the four conserved amino acids (see Additional file 4 and 5). If a new motif was not referred in the 18 groups, we modified our Perl program with the new motif to search against the entire proteome again. For instance, when a detected C-X_8_-C-X_6_-C-X_3_-H motif was not mentioned by Berg et al., we rewrote new regular expression for matching the sequences of this motif. After verifying the reliability of the new motif in the protein by SMART and Pfam, we added it to the CCCH family. The motifs which were not mentioned by Berg et al. were also listed in Table 1, making a total of 23 groups.

Community microarray data, Massively Parallel Signature Sequencing searches, and ESTs database provided confirmative transcript information for all 68 genes (Table 2). From the above evidences, no pseudogene was found among the 68 genes. The CCCH genes were listed in Table 2 and their entry numbers were assigned according to the order of CCCH genes appearing on the Arabidopsis chromosomes, from the short arm to the long arm, and from chromosomes 1 to 5 (see Additional file 6).

**Table 2:** The CCCH gene family of Arabidopsis.

**Gene name**	**Gene Identifier**	Expression*	**Number of CCCH motif**	**Size (aa)**	**Mass (Da)**
AtC3H2	AT1G03790	ABC	2	393	43614.4
AtC3H3	AT1G04990	ABC	5	404	44704.5
AtC3H4	AT1G07360	ABC	1	481	53588.0
AtC3H5	AT1G10320	ABC	2	757	89065.0
AtC3H6	AT1G19860	ABC	1	413	45127.0
AtC3H7	AT1G21570	ABC	5	658	74969.4
AtC3H8	AT1G27650	ABC	2	296	34572.3
AtC3H9	AT1G29570	AC	1	321	39645.6
AtC3H10	AT1G29600	BC	2	287	33676.2
AtC3H11	AT1G30460	ABC	3	678	75026.7
AtC3H12	AT1G32360	ABC	3	384	40706.1
AtC3H13	AT1G48195	AB	2	83	9451.0
AtC3H14	AT1G66810	ABC	2	310	35108.7
AtC3H15	AT1G68200	ABC	2	308	34227.4
AtC3H16	AT1G75340	ABC	1	486	52666.2
AtC3H17	AT2G02160	ABC	3	669	75831.5
AtC3H18	AT2G05160	ABC	1	536	61349.4
AtC3H19	AT2G16470	AB	1	670	72185.1
AtC3H20	AT2G19810	ABC	2	359	39845.4
AtC3H21	AT2G20280	ABC	1	371	42389.1
AtC3H22	AT2G24830	ABC	1	497	55935.1
AtC3H23	AT2G25900	ABC	2	315	35464.6
AtC3H24	AT2G28450	ABC	1	809	89022.8
AtC3H25	AT2G29580	ABC	1	483	54252.5
AtC3H26	AT2G32930	BC	5	453	49747.4
AtC3H27	AT2G33835	AB	1	587	64376.0
AtC3H28	AT2G35430	BC	2	180	19991.1
AtC3H29	AT2G40140	ABC	2	597	66153.4
AtC3H30	AT2G41900	ABC	2	716	77992.2
AtC3H31	AT2G47680	ABC	2	1015	115085.0
AtC3H32	AT2G47850	ABC	5	553	59273.7
AtC3H33	AT3G02830	ABC	5	397	44181.6
AtC3H34	AT3G06410	ABC	5	437	46195.1
AtC3H35	AT3G08505	AB	4	323	36563.0
AtC3H36	AT3G12130	ABC	2	248	25952.2
AtC3H37	AT3G12680	ABC	6	341	37693.5
AtC3H38	AT3G18640	ABC	1	676	75581.4
AtC3H39	AT3G19360	ABC	3	386	43121.5
AtC3H40	AT3G21810	ABC	1	388	45072.2
AtC3H41	AT3G27700	ABC	1	908	99228.3
AtC3H42	AT3G47120	ABC	2	352	40684.3
AtC3H43	AT3G48440	ABC	5	448	49864.0
AtC3H44	AT3G51120	ABC	1	1247	137478.0
AtC3H45	AT3G51180	ABC	1	521	57369.9
AtC3H46	AT3G51950	ABC	1	540	59789.3
AtC3H47	AT3G55980	ABC	2	580	64058.4
AtC3H48	AT4G25440	AB	2	430	46662.0
AtC3H49	AT4G29190	ABC	2	356	40264.4
AtC3H50	AT4G38890	ABC	1	700	78569.5
AtC3H51	AT5G06420	ABC	1	378	42460.1
AtC3H52	AT5G06770	ABC	2	240	25367.7
AtC3H53	AT5G07060	C	1	363	41754.6
AtC3H54	AT5G07500	AC	2	245	28175.4
AtC3H55	AT5G12440	ABC	1	552	61459.2
AtC3H56	AT5G12850	ABC	2	706	77775.4
AtC3H57	AT5G16540	ABC	5	368	40792.9
AtC3H58	AT5G18550	ABC	5	456	48392.7
AtC3H59	AT5G40880	ABC	1	472	51297.8
AtC3H60	AT5G42820	ABC	2	283	33231.9
AtC3H61	AT5G44260	ABC	2	381	42057.7
AtC3H62	AT5G49200	ABC	1	419	46069.8
AtC3H63	AT5G51980	ABC	2	437	47287.9
AtC3H64	AT5G56900	ABC	2	596	66421.4
AtC3H65	AT5G56930	B	3	675	73844.5
AtC3H66	AT5G58620	ABC	2	607	66447.7
AtC3H67	AT5G63260	ABC	5	435	48606.6
AtC3H68	AT5G66270	AB	1	449	49442.5

*The evidence for gene expression by (A) EST clones, (B) massively parallel signature sequencing (MPSS) and (C) community microarray data.

### Phylogenetic analysis of the CCCH proteins in Arabidopsis

**Figure 3:**
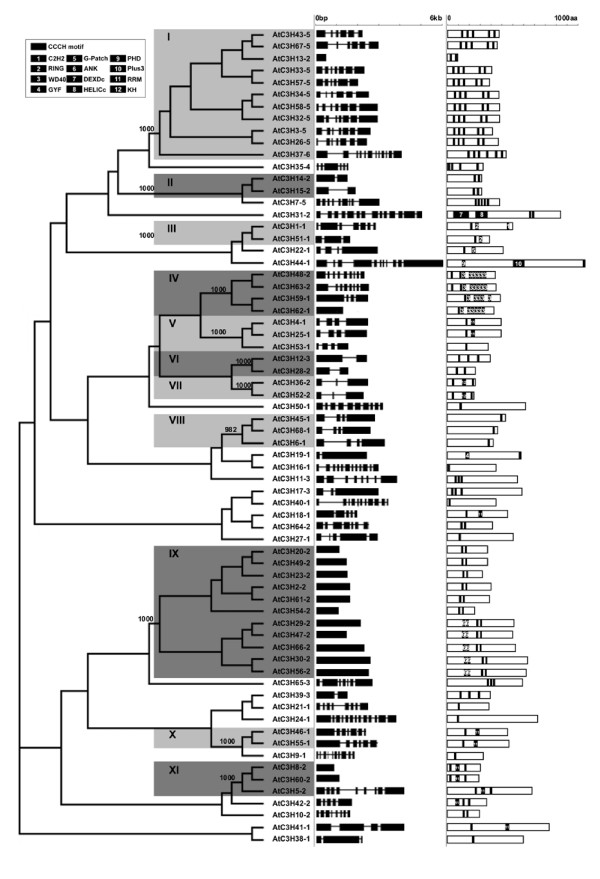
**An analytical view of the Arabidopsis CCCH gene family**. The following parts are shown from left to right. Protein neighbor-joining tree: The unrooted tree, constructed using ClustalX (1.83), summarizes the evolutionary relationship among the 68 members of CCCH families. The neighbor-joining tree was constructed using aligned full-length amino acid sequences. The proteins are named according to their gene name (see Table 2) with the CCCH zinc finger number of each protein. The tree shows the 11 major phylogenetic subfamilies (left column, numbered I to XI and marked with different alternating tones of a gray background to make subfamily identification easier) with high predictive value. The numbers beside the branches represent bootstrap values (≥500) based on 1000 replications that were used to class the major 11 subfamilies. Gene structure: The gene structure is presented by black exon(s) and spaces between the black boxes correspond to introns. The sizes of exons and introns can be estimated using the horizontal lines. Protein structure: Each black box represents the motif in the protein, as indicated in the table on the left side. The conserved motifs outside CCCH motif are highlighted with an arranged number, and the same number referred to the same motif. The length of the motif can be estimated using the scale at top. aa, amino acids.

To evaluate the evolutionary relationships within the CCCH gene family in Arabidopsis, we performed a phylogenetic analysis of the 68 Arabidopsis protein sequences to construct a phylogenetic tree (Figure 3). Although the signature CCCH motif of the CCCH proteins is well conserved, the motif number of each protein and spacing amino acids between adjacent CCCH zinc-finger motifs are diverse, so it is difficult to make alignment using CCCH motifs. Accordingly, an alignment of full-length CCCH proteins was constructed using ClustalX and refined manually [41]. A phylogenetic tree was generated with the neighbor-joining method [42]. For statistical reliability, we conducted bootstrap analysis with 1000 replicates. The tree topologies are similar with different tree-building methods except in deep nodes. From the values obtained in the bootstrap analysis, it is apparent that the phylogenetic relationship is unclear and the bootstrap values are low in deep nodes. Nevertheless, in outer clades, the CCCH protein has better resolution, permitting subfamilies of proteins to be delimited. This phenomenon was also observed in the analysis of the bHLH [43], Dof [8], WRKY [44], and IQD [45] transcription factor families. Alternatively, it may be due to the divergence of the CCCH motif, especially the diverse motif numbers and spacing in the amino acid sequences between Cys and His in each protein. We could not infer evolutionary relationships between the different subfamilies of CCCH proteins because the internal nodes did not show high support. By contrast, within each subfamily, the strong amino acid sequence conservation is evident from the short branch lengths at the tips of the tress, suggestive of strong evolutionary relationships among subfamily members.

Based on the statistical support of each branch, we divided the Arabidopsis CCCH family into 11 subfamilies, which was supported by more than 90% bootstrap value, designated I to XI (Figure 3). The genes with low bootstrap value were not divided into subfamilies, and also were not taken into consideration for further analysis. The genes clustered pairwisely and supported by bootstrap value 1000 are mostly paralogous genes in Arabidopsis. Totally, 18 pairs of putative paralogous genes were found in Arabidopsis.

### The analysis of protein motif and gene structure

To discover motifs shared among related proteins within the family, we used MEME [46], which performs motif searches in groups of related DNA or protein sequences. The search was performed separately for each of the subfamilies of proteins. Moreover, SMART and Pfam were used to annotate the motifs found by MEME. As expected, the proteins within same subfamily exhibit the common CCCH motifs, suggesting the major functional role of CCCH motif in these proteins, although few motifs outside the CCCH domain were detected by MEME as well. The schemes of the protein motifs of the individual members of the CCCH family clearly demonstrate structural similarities among the proteins within one subfamily (Figure 3). For example, the members of subfamily IV contain tandem WD40 domains. All eleven members of subfamily IX consist of three highly conserved tandem motifs (C-X_5_-H-X_4_-C-X_3_-H, C-X_7–8_-C-X_5_-C-X_3_-H and C-X_5_-C-X_4_-C-X_3_-H) and nine members of subfamily I contain five C-X_8_-C-X_5_-C-X_3_-H zinc finger motifs. Furthermore, twelve proteins contain well-defined RNA-binding domain RRM or KH motifs suggesting their potential roles involved in RNA binding [47].

The gene structures in terms of intron number and gene length were also consistent with the phylogenetic subfamilies defined in Figure 3. Most members in same subfamilies had similar intron/exon structure. The fact that they not only contain common motifs but also share very similar intron/exon structure supports their close evolutionary relationship and membership in the same subfamily. Taken together, our results validate the classification of the CCCH family genes of Arabidopsis.

### Evolution and divergence of the CCCH family genes in Arabidopsis

**Figure 4:**
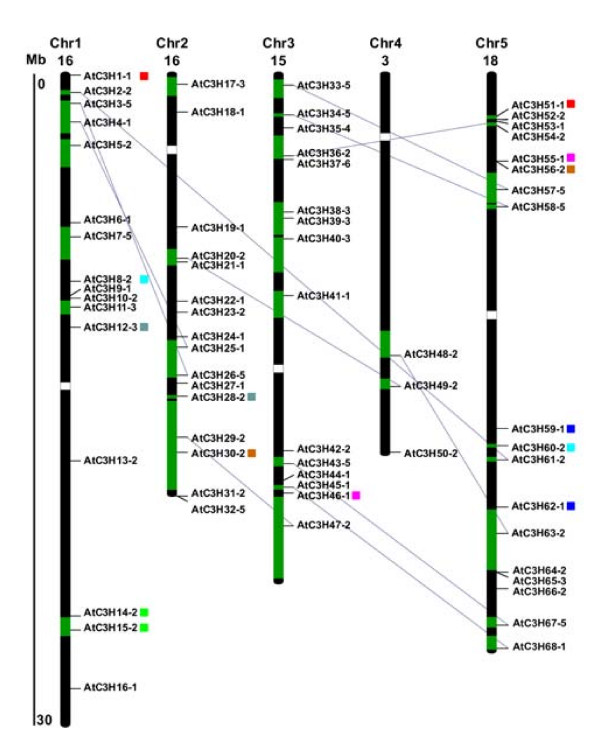
**Chromosomal distribution and segmental duplication events for Arabidopsis CCCH genes**. The chromosome number is indicated at the top of each chromosome. The green boxes indicate the duplicated segmental regions resulting from the most recent polyploidy. Only the duplicated regions containing CCCH genes are shown. Blue lines connect corresponding sister gene pairs in duplicated blocks. AtC3H1 and AtC3H51, AtC3H8 and AtC3H60, AtC3H12 and AtC3H28, AtC3H14 and AtC3H15, AtC3H30 and AtC3H56, AtC3H46 and AtC3H55, AtC3H59 and AtC3H62 are potential duplicated gene pairs which are marked with the same color rectangle, as described in the text.

The Arabidopsis genome has undergone several rounds of genome-wide duplication events, including polyploidy [48,49], which has great impact on the amplification of members of a gene family in the genome. To further investigate the relationship between the genetic divergence within the CCCH family and gene duplication in Arabidopsis, the chromosomal location of each CCCH gene was determined from the genomic sequences of Arabidopsis [50]. Based on the chromosomal location information provided by the NCBI [51] and TAIR [52], we localized 68 CCCH genes in Arabidopsis chromosomes and determined that the genes are distributed across all five chromosomes (Figure 4). Relatively low densities of CCCH genes were observed in some chromosomal regions, including the top of chromosome 4, and the bottom of chromosome 1. Twenty-two genes were found in previously identified duplicated segmental regions on chromosomes that are the result of a polyploidy that occurred around 24 to 40 million years ago, probably close to the emergence of the crucifer family [53]. Another seven gene pairs (AtC3H1 and AtC3H51, AtC3H8 and AtC3H60, AtC3H12 and AtC3H28, AtC3H14 and AtC3H15, AtC3H30 and AtC3H56, AtC3H46 and AtC3H55, AtC3H59 and AtC3H62) were identified to share common gene structure and zinc finger motif. Although Blanc et al. did not include the seven gene pairs as duplicated pair genes in recently duplicated segmental chromosomes, the phylogenetic relationship and sequence similarity suggest that they are closely related to each other. Furthermore, AtC3H12 and AtC3H28 were listed as duplicated genes in the segmental duplications dataset maintained from TIGR. Therefore, they were considered to be putative duplicated genes (Figure 4). Consequently, about 53% of CCCH genes, which lie within recently duplicated segmental chromosomes, have a clear relative in these regions. Since the density of the duplicated genes in recently duplicated segmental chromosomes was reported to be 28.0% ± 7.8% [53], the duplicated pairs of CCCH genes have been preferentially retained compared with other genes. This finding is consistent with a previous study demonstrating that duplicated genes involved in signal transduction and transcription are preferentially retained [54,55]. In summary, large-scale segmental duplication events appear to have exclusively contributed to the current complexes of the CCCH gene family.

In order to test whether CCCH family is ubiquitous in other organisms, CCCH genes in moss were also identified. Although ESTs are incomplete, a preliminary examination using the BLAST program suggested that almost all of the CCCH genes belonging to subfamily I, II, III, IV, V, VI, VII, X and XI in Arabidopsis have corresponding homologous members in moss. For subfamily VIII, since only 3 members are present in Arabidopsis, it is difficult to detect their corresponding homologous genes in the incomplete EST database of moss. However, the subfamily IX, which contains 11 genes in Arabidopsis, is not extracted in moss, suggesting that it is a particular subfamily only belonging to advanced plants or arises from the evolutionary process to adapt the circumstance.

To explore the evolutionary history of the CCCH gene family in greater detail and to testify our assumption about subfamily IX, we searched publicly available genomic and EST databases for all angiosperm species represented in the TIGR Plant Gene Indices as well as for the gymnosperm *Pinus ssp*. [56]. As expected, almost all of the members in Arabidopsis have their counterparters in these higher plants. These results suggest that the basis of the phylogenetic topology of the CCCH family had already been established before the divergence of vascular plants and the subfamily IX may be a special subfamily belonging only to higher plant.

### Identification of CCCH genes in rice

Rice is one of the most important food crops in the world and it has been used as a major model species in plant (especially monocot) functional genomics research because of its relatively small genome size and extensively sequenced genome [32]. To explore the occurrence and size of the CCCH gene family in rice, we wrote a Perl program to analyze the genome file of rice (*ssp. japonica*) got from The TIGR Rice Genome Annotation Database and Resource (see Additional file 7). Multiple BLAST searches were also performed in several rice databases using the typical CCCH protein sequence as the queries to validate the primary results. After detecting the CCCH motifs by SMART and Pfam, 67 CCCH genes were identified in rice (Table 3, see Additional file 8). To determine their phylogenetic relationships, a phylogenetic tree was constructed using the full length protein sequences. Like the CCCH protein family in Arabidopsis, the phylogenetic analysis reveals 8 subfamilies supported by high bootstrap values. The members of same subfamily share the similar gene structure and zinc finger motifs (see Additional file 9A). We also detected two large subfamilies in the tree, in which the members share the same characteristic with the genes of corresponding subfamilies in Arabidopsis. Unlike the Arabidopsis CCCH family, the distribution of CCCH genes in the rice genome is clearly biased towards the chromosomes 1 to 7 (see Additional file 10). Four CCCH genes are each found on chromosomes 8, 9 and 12, and only one gene, however, is present on chromosome 10 and 11, respectively. The topology of the phylogenetic tree of rice CCCH genes indicates 13 pairs of putative duplicated genes that are close paralogs (OsC3H2 and OsC3H35, OsC3H5 and OsC3H6, OsC3H9 and OsC3H39, OsC3H10 and OsC3H37, OsC3H14 and OsC3H44, OsC3H19 and OsC3H32, OsC3H22 and OsC3H23, OsC3H24 and OsC3H67, OsC3H38 and OsC3H60, OsC3H40 and OsC3H49, OsC3H42 and OsC3H43, OsC3H54 and OsC3H51, OsC3H63 and OsC3H66). These genes represent 39% of the rice CCCH gene family members and might have evolved from putative rice genome duplication events.

**Table 3:** The CCCH gene family of rice.

**Gene name**	**LOC NO**.	**Number of CCCH motif**
OsC3H1	LOC_Os01g07930	2
OsC3H2	LOC_Os01g09620	2
OsC3H3	LOC_Os01g14870	1
OsC3H4	LOC_Os01g15300	2
OsC3H5	LOC_Os01g15350	5
OsC3H6	LOC_Os01g15460	5
OsC3H7	LOC_Os01g39100	3
OsC3H8	LOC_Os01g42970	6
OsC3H9	LOC_Os01g45730	2
OsC3H10	LOC_Os01g53650	2
OsC3H11	LOC_Os01g61830	1
OsC3H12	LOC_Os01g68860	5
OsC3H13	LOC_Os02g06584	1
OsC3H14	LOC_Os02g10080	3
OsC3H15	LOC_Os02g19804	1
OsC3H16	LOC_Os02g35150	2
OsC3H17	LOC_Os02g45480	2
OsC3H18	LOC_Os02g55000	1
OsC3H19	LOC_Os02g58440	3
OsC3H20	LOC_Os03g02160	1
OsC3H21	LOC_Os03g18950	2
OsC3H22	LOC_Os03g21140	1
OsC3H23	LOC_Os03g21160	1
OsC3H24	LOC_Os03g49170	2
OsC3H25	LOC_Os03g61110	1
OsC3H26	LOC_Os04g02730	1
OsC3H27	LOC_Os04g32340	1
OsC3H28	LOC_Os04g35800	6
OsC3H29	LOC_Os04g41060	1
OsC3H30	LOC_Os04g56750	1
OsC3H31	LOC_Os04g57010	3
OsC3H32	LOC_Os04g57600	3
OsC3H33	LOC_Os05g03760	2
OsC3H34	LOC_Os05g08400	3
OsC3H35	LOC_Os05g10670	2
OsC3H36	LOC_Os05g41790	1
OsC3H37	LOC_Os05g45020	2
OsC3H38	LOC_Os05g48960	2
OsC3H39	LOC_Os05g50080	2
OsC3H40	LOC_Os06g07350	1
OsC3H41	LOC_Os06g21390	4
OsC3H42	LOC_Os06g32720	3
OsC3H43	LOC_Os06g32860	3
OsC3H44	LOC_Os06g41390	2
OsC3H45	LOC_Os06g46400	3
OsC3H46	LOC_Os06g49080	1
OsC3H47	LOC_Os07g04580	2
OsC3H48	LOC_Os07g04650	3
OsC3H49	LOC_Os07g18050	1
OsC3H50	LOC_Os07g38090	2
OsC3H51	LOC_Os07g39440	1
OsC3H52	LOC_Os07g47240	2
OsC3H53	LOC_Os07g48410	1
OsC3H54	LOC_Os08g03310	1
OsC3H55	LOC_Os08g04170	3
OsC3H56	LOC_Os08g06330	3
OsC3H57	LOC_Os08g38370	2
OsC3H58	LOC_Os09g13530	1
OsC3H59	LOC_Os09g19940	2
OsC3H60	LOC_Os09g31482	2
OsC3H61	LOC_Os09g36090	1
OsC3H62	LOC_Os10g25220	1
OsC3H63	LOC_Os11g28270	5
OsC3H64	LOC_Os12g03554	1
OsC3H65	LOC_Os12g18120	5
OsC3H66	LOC_Os12g21700	5
OsC3H67	LOC_Os12g33090	2

### Comparative phylogenetic analyses of the CCCH genes in Arabidopsis and rice

In order to evaluate the evolutionary relationship among the CCCH proteins, we performed a phylogenetic analysis based on the full-length amino acid sequences of Arabidopsis and rice. Because of the large number of taxa and relatively small number of characters in full-length sequences, the bootstrap values of internal nodes were low, whereas the outer nodes had more credible bootstrap values, allowing for clustering of the CCCH genes of Arabidopsis and rice into 20 subfamilies (see Additional file 9B). The tree topology, as well as the subfamily organization, resembled those from the rice and Arabidopsis individual trees. Eight subfamilies from Arabidopsis and rice clustered together into the same branch in the combined tree and 30 putative orthologs were identified in the tree (see Additional file 9B), suggesting that an ancestral set of CCCH genes already existed before the monocot-eudicot divergence. Moreover, the paralogous genes of Arabidopsis and rice were already displayed as paralogs in the respective trees.

**Figure 5:**
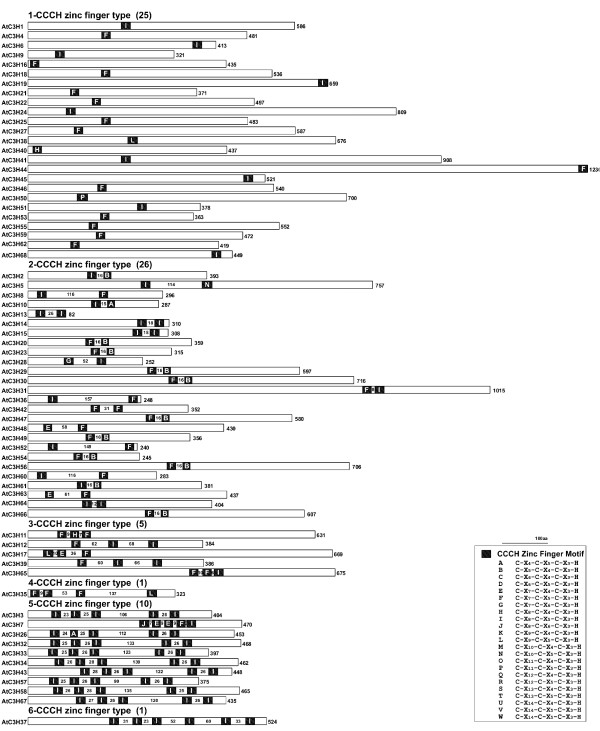
**Schematic structures of Arabidopsis CCCH proteins**. The figure is schematic protein structures of all CCCH-type zinc-finger proteins identified in Arabidopsis. The CCCH zinc fingers are shown by black boxes. Types of zinc fingers are indicated by A to W, which are cross-indexed in the table on the right side. Numbers of amino acids in the spacers are indicated on each spacer region for several-fingered proteins. The lengths of each protein are shown on the right of the schematic structures. Numbers of different types of CCCH zinc finger proteins are presented in the brackets.

Despite the differences in genome sizes between Arabidopsis and rice (125 Mb and 389 Mb respectively) and encoded number of genes, the two plant species appear to have a very similar number of genes encoding CCCH zinc-finger proteins (68 and 67 putative numbers, respectively). Compared with other gene families in Arabidopsis and rice, the CCCH gene family is one of the largest families in plant and includes diverse members with distinct specificities. As shown in Figure 5, the proteins containing C-X_7–8_-C-X_5_-C-X_3_-H motif constitute the largest groups in the CCCH zinc-finger family. In Arabidopsis, 42 members contain C-X_7_-C-X_5_-C-X_3_-H motif and 44 members contain C-X_8_-C-X_5_-C-X_3_-H motif, while the number is 35 and 36 in rice, respectively, suggesting that C-X_7–8_-C-X_5_-C-X_3_-H motif may be an ancestor of other CCCH motifs. In addition, five novel CCCH motifs beyond the 18 groups were identified, including C-X_4_-C-X_5_-C-X_3_-H, C-X_5_-C-X_4_-C-X_3_-H, C-X_7_-C-X_6_-C-X_3_-H, C-X_8_-C-X_6_-C-X_3_-H and C-X_15_-C-X_5_-C-X_3_-H. Further experiments are required to determine their binding activity to Zn^2+ ^and biological functions.

**Figure 6:**
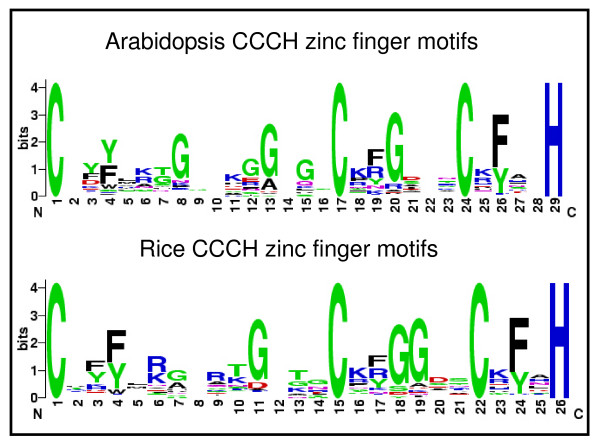
**Sequence logos for the CCCH zinc finger motifs of Arabidopsis and rice**. Numbers on the x-axis represent the sequence positions in zinc finger motifs. The y-axis represents the information content measured in bits. The sequence logos were derived using WebLogo [4].

To determine sequence features of these CCCH motifs, we performed sequence alignments of 302 CCCH motifs (152 from Arabidopsis and 150 from rice) using the Clustal X program [41]. The weblogo indicated that four (three cysteines and one histidine) amino acids are completely conserved among all the CCCH motifs and more than 75% of the CCCH motifs contain glycine and phenylalanine (Figure 6) [57]. In addition, the distributions of conserved amino acids among the CCCH motifs of both Arabidopsis and rice are similar. Given these characteristic of verified protein sequences, we developed a new criteria to objectively define those sequences to be considered as CCCH motif. The CCCH proteins are characterized by one to six C-X_4–15_-C-X_4–6_-C-X_3_-H motifs which are glycine-rich and phenylalanine-rich sequences.

### The expression pattern of CCCH genes in Arabidopsis and rice

**Figure 7:**
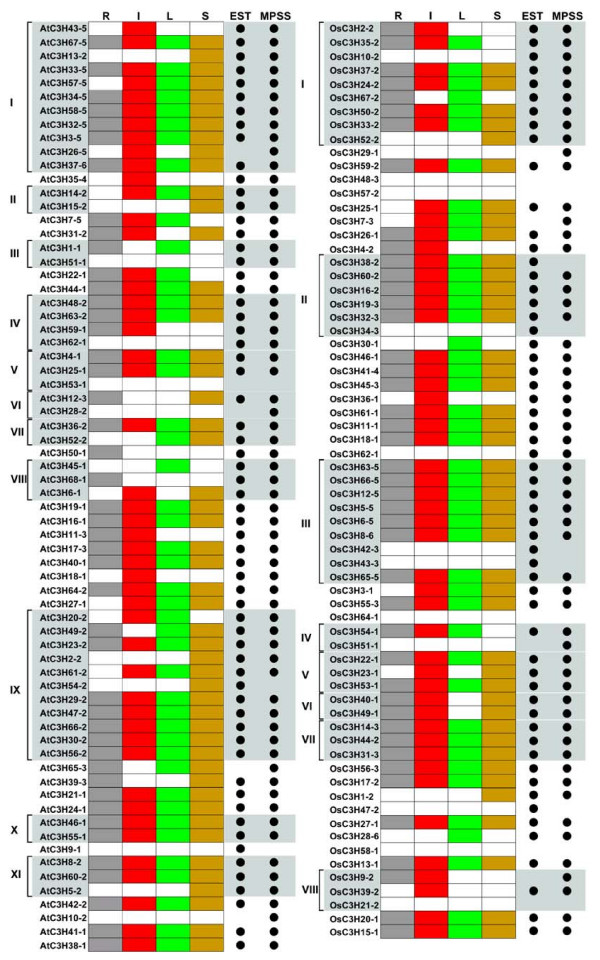
**The expression patterns for Arabidopsis and rice CCCH genes from MPSS and EST data**. The expression patterns for Arabidopsis (the part of left side) and rice (the part of right side) CCCH genes are shown. The letter R above the column of expression data refers to root, I refers to inflorescence, L refers to leaf, and S refers to seed (silique). A positive signal is indicated by a colored box for the following tissues: grey for roots (R), red for inflorescences (I), green for rosette leaves (L), and yellow for siliques (S). The white box indicates that no expression could be detected. The number on the left indicates the subfamily and the black points on the right show the origin of expression data for each gene. CCCH proteins are aligned in the same order as they appear in the phylogenetic trees. Subfamilies of CCCH proteins are highlighted by vertical bars next to the gene identifiers. The expression profile of AtC3H37 (*HUA1*) was got from RNA filter hybridization [19].

Since gene expression patterns can provide important clues for gene function, we examined the expression of Arabidopsis and rice CCCH genes in root, leaf, flower and seed tissues using massively parallel signature sequencing (MPSS) data and EST data of NCBI (Figure 7) [58,59]. In Arabidopsis, expression profiles of 65 and 63 CCCH genes were extracted from MPSS and EST databases, respectively. After integrating two data together, we found that most of the genes have a very broad expression spectrum, and only six genes (AtC3H9, AtC3H10, AtC3H28, AtC3H51, AtC3H53 and AtC3H62) were not detected from any specific tissue according to EST and MPSS data. Except AtC3H53, five of these six genes were detected in mix tissue of Arabidopsis from EST database. Furthermore, we also summarized the expression of rice CCCH genes using the same methods. Expression of 57 rice CCCH genes was detected from MPSS database, and 58 had matching ESTs. Thirteen CCCH genes in rice had no tissue expression pattern, while eight of them contain corresponding ESTs and five (OsC3H21, OsC3H48, OsC3H57, OsC3H58 and OsC3H64) without expression information might be pseudogenes, or expressed at specific developmental stages or under special conditions.

By combining the EST and MPSS results together, we found that the accumulation of CCCH gene transcripts not only is associated with different tissues, but also the expression pattern of each CCCH gene member differed. According to expression profiles, CCCH genes can be classified into three groups. The largest group is the genes that expressed in all tissues, including 33 genes in Arabidopsis and 36 genes in rice, and the second group contains 18 Arabidopsis CCCH genes and 12 rice CCCH genes whose expression were detected in the majority, but not all, of the tissues. Interestingly, the third group includes 17 genes with very specific expression. AtC3H50 and AtC3H68 are exclusively expressed in roots, while AtC3H18, AtC3H35, AtC3H43, OsC3H36 and OsC3H39 in inflorescences, AtC3H45, OsC3H28 and OsC3H30 in leaves, AtC3H2, AtC3H5, AtC3H13, AtC3H15, AtC3H54, OsC3H1 and OsC3H52 in seeds. When compared the expression pattern of the AtC3H45, which could not be detected by SMART and Pfam but contains a C-X_8_-C-X_5_-C-X_3_-H motif, with its sister gene AtC3H68, we found that they share different expression profiles with relatively low level (AtC3H45 could be only detected in leaves, while AtC3H68 is exclusively expressed in roots). In addition, we examined the expression of other CCCH duplicated gene pairs of both Arabidopsis and rice, and only 20 of 31 pairs (18 duplicated gene pairs in Arabidopsis and 13 in rice) share the same expression pattern. These results are consistent with the previous research by Blanc and Wolfe, that the expression profiles of the two paralogs have diverged in concert, forming two parallel networks, and the expression of each gene is strongly correlated with the other nonhomologous genes in its network but poorly correlated with its paralog in the other network, suggesting functional diversification of the surviving duplicated genes is a major feature of the long-term evolution of polyploids [55].

Expression profiling within different tissues is only the first step to understanding the function of CCCH genes. Overall, the CCCH family members show diverse expression patterns. A majority of the CCCH genes are expressed in all tissues tested, with various expression levels. It might be a common character of large transcription factor families, such as MYB family [60]. In addition, the genes with specific expression patterns can be the focus of functional studies for their possible roles in specific tissues. Because of the limitation of current information from MPSS and EST databases, expression of some genes within the tissues might not be detected. Further investigation of spatial and developmental expression patterns will need to be performed to obtain more detailed expression information.

### Characteristics of subfamily IX genes of Arabidopsis and subfamily I genes of rice

**Figure 8:**
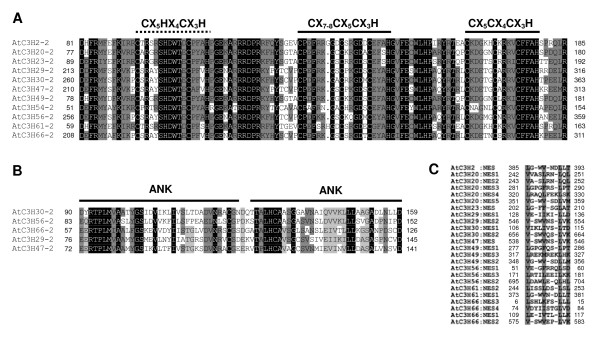
**Amino acid sequence analysis of the members in subfamily IX of Arabidopsis**. A, Multiple sequence alignment of the three zinc finger motifs of the members in the subfamily IX. The black and dashed bars represent CCCH motifs and putative CHCH zinc finger, respectively. Black and gray shading indicate identical and conserved amino acid residues present in more than 50% of the aligned sequences, respectively. B, Amino acid sequence alignment of ankyrin repeats in CCCH family. C, Amino acid sequence alignment of putative NES sequences in subfamily IX.

Subfamily IX containing 11 members in Arabidopsis and subfamily I containing 9 members in rice are the largest CCCH subfamily in each species, respectively. All the proteins encoded by these 20 genes include two CCCH motifs. With the exception of OsC3H10, OsC3H37 and OsC3H52 in rice, the products of 17 genes commonly contain two tandem consensus motifs, C-X_7–8_-C-X_5_-C-X_3_-H and C-X_5_-C-X_4_-C-X_3_-H. In OsC3H10 and OsC3H37 proteins, the first zinc finger is replaced by a C-X_10_-C-X_5_-C-X_3_-H motif, while in OsC3H52 the first zinc finger is C-X_15_-C-X_5_-C-X_3_-H. Amino acid sequence analysis revealed that all twenty members contain highly conserved C-X_5_-C-X_4_-C-X_3_-H motif, which is a characteristic of these proteins. Additionally, putative consensus CHCH (C-X_5_-H-X_4_-C-X_3_-H) motif was also observed in these 20 proteins, implying that it may be a novel zinc finger motif and execute some biological functions (Figure 8A). Moreover, the number of amino acids between the three motifs is invariable and all the 20 genes have no introns (Figure 3, see Additional file 9A).

**Figure 9:**
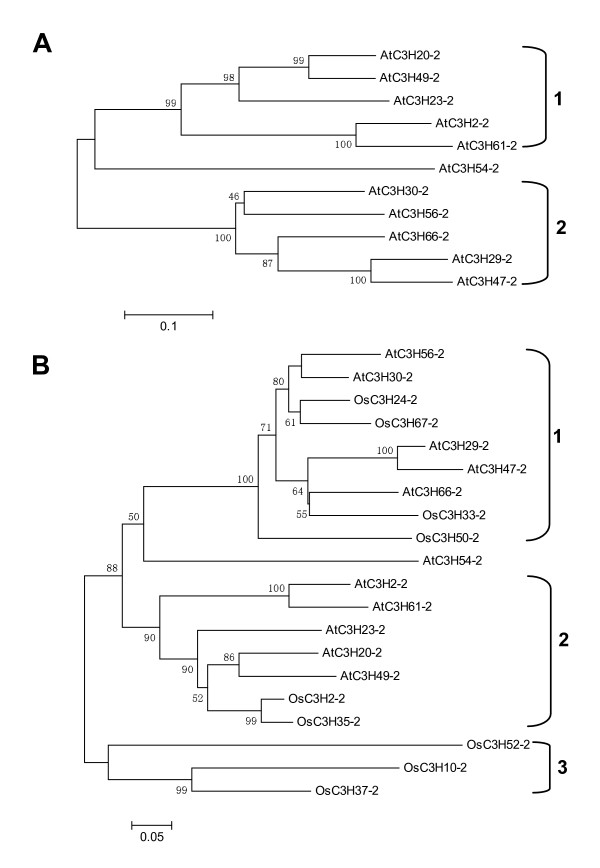
**Phylogenetic trees of genes in subfamily IX of Arabidopsis and subfamily I of rice**. A, Phylogenetic tree of Arabidopsis CCCH genes in subfamily IX. The unrooted tree was inferred by MEGA 3.1 and the neighbor-joining method after the alignment of the full-length amino acid sequences of the 11 Arabidopsis genes in subfamily IX. The numbers beside the branches represent bootstrap values based on 1000 replications. Subgroups of CCCH proteins are highlighted by vertical bars next to the gene identifiers. The scale bar corresponds to 0.1 estimated amino acid substitutions per site. B, Joined phylogenetic tree of rice subfamily I and Arabidopsis subfamily IX CCCH genes. The unrooted tree was constructed using MEGA 3.1 and the neighbor-joining method after the alignment of the full-length amino acid sequences of 20 Arabidopsis and rice genes. Numbers on branches indicate the percentage of 1000 bootstrap replicates that support the adjacent node. Black braces and numerals at right indicate the three subgroups. The scale bar corresponds to 0.05 estimated amino acid substitutions per site.

As shown in Figure 9A, phylogenetic analysis indicated that the subfamily IX of Arabidopsis consists of two major subgroups. Interestingly, with exception of two CCCH motifs, ankyrin (ANK) repeat motif was also identified by MEME search using amino acid sequences of all members of subgroup 2 (Figure 8B). The ANK repeat motif is one of the most common protein-protein interaction motifs in nature, and it has been found in proteins of diverse function such as transcriptional initiators, ion transporters and signal transducers [61-64]. The database search further revealed that only six genes coding proteins with ANK repeats and zinc finger domains in whole Arabidopsis genome, five of them belong to CCCH subfamily IX. In addition, to evaluate the evolutionary relationships within the 20 genes, we performed a combined phylogenetic analysis of the 20 Arabidopsis and rice amino acid sequences to obtain a joint tree. Three subgroups can be observed from the combined tree (Figure 9B). In subgroup 1 of the combined tree, all nine genes of both Arabidopsis and rice contain the ANK domain. Surprisingly, within each subgroup, the rice and Arabidopsis genes appear closely related to genes of the same species, and only one putative ortholog pairs (AtC3H66 and OsC3H33) were identified in the combined tree. One possible explanation of this phenomenon is that these genes may go through a complex and extensive evolution in each species after monocot-eudicot divergence.

Previous studies demonstrated that OsDOS, AtCPSF30 and HUA1 are nuclear-localized proteins [19,20,30]. The database research with full-length protein sequences of subfamily IX of Arabidopsis and subfamily I of rice suggested that all members are localized in the cell nucleus (see Methods). In animal, TTP was shown to export from the nucleus mediated by a Leucine-rich Nuclear Export Signal (NES). To our knowledge, no Leucine-rich NES in plant proteins has been identified. When we performed our program using the developed widely accepted NES consensus [LV]-x(2,3)-[LIVFM]-x(2,3)-L-x-[LIMTKD] to detect 68 Arabidopsis CCCH proteins [65], 54 proteins containing putative NES sequences were identified including all of 11 members of subfamily IX (Figure 8C, see Additional file 11 and 12). The result suggests that all subfamily IX proteins of Arabidopsis may be nucleocytoplasmic shuttle proteins involved in signal transduction events [66].

**Figure 10:**
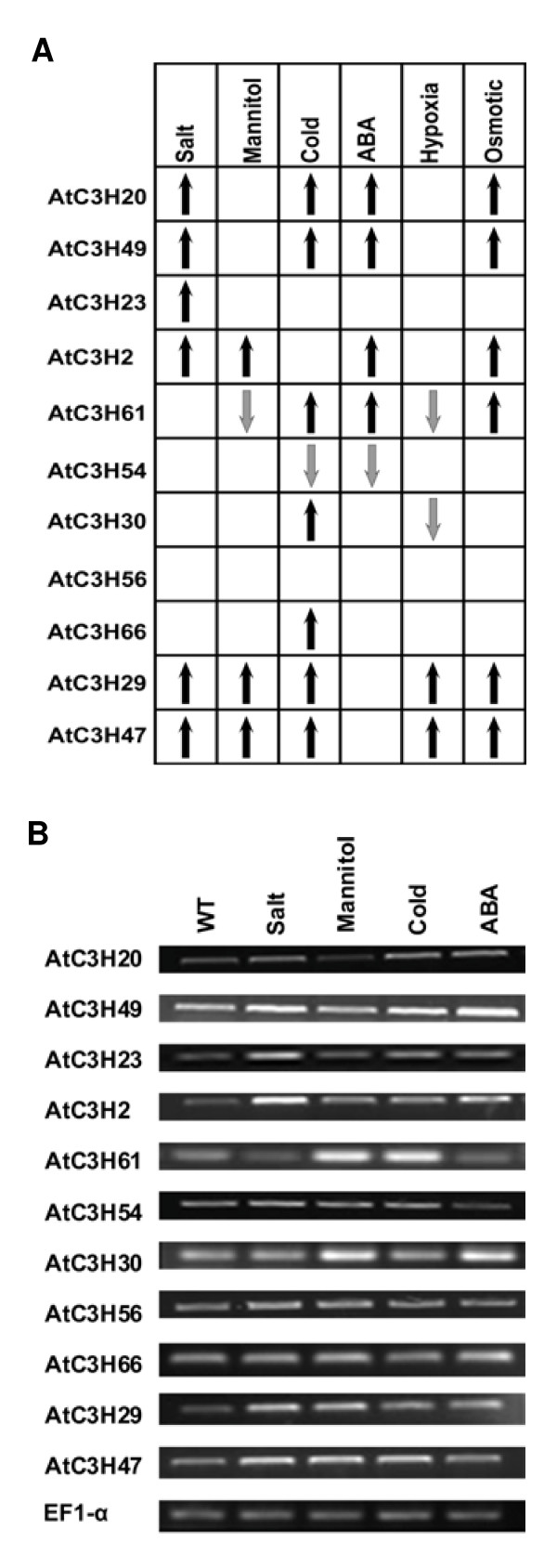
**The expression patterns of subfamily IX genes in response to abiotic stress and ABA treatments**. A, Microarray data for the CCCH genes of subfamily IX of Arabidopsis were extracted using Genevestigator. Up- and down-expression patterns of eleven genes under six different treatments are indicated by black and grey arrowhead, respectively. B, Expression pattern for genes in subfamily IX of Arabidopsis following stress and ABA treatments detected by RT-PCR. Plants were grown on Murashige and Skoog (1962) (MS) agar medium for 3 weeks and were treated with NaCl (300 mM), mannitol (300 mM), cold (4°C), ABA (100 μM) or water (as a control). The EF1-α gene was used as an internal control.

Recently, a few genes within these 20 genes have been shown to play crucial roles in abiotic and/or biotic stress-responsive gene expression. *OsDOS *(LOC_Os01g09620) was proved to be involved in JA pathway [30]. *ZFAR1 *(At2g40140) transcript was induced by Botrytis in inoculated Arabidopsis lines and *zfar1 *mutant was hypersensitive to ABA [67]. In order to better understand the function of these genes, we firstly examined the expression of all the genes of subfamily IX of Arabidopsis in response to multiple environmental stimuli by means of microarray data available at Genevestigator site [68]. The results revealed that all genes of subfamily IX were activated or suppressed by various stresses, including salt, cold, mannitol, ABA, hypoxia and osmotic stress (Figure 10A). Secondly, we analyzed the expression of these 11 genes in Arabidopsis plants treated with drought (mannitol), salt, cold, and ABA by RT-PCR. As shown in Figure 10B, the RT-PCR results are in better agreement with the microarray data, suggesting their involvement in abiotic and/or biotic stress.

### RNA-binding roles for CCCH proteins

**Figure 11:**
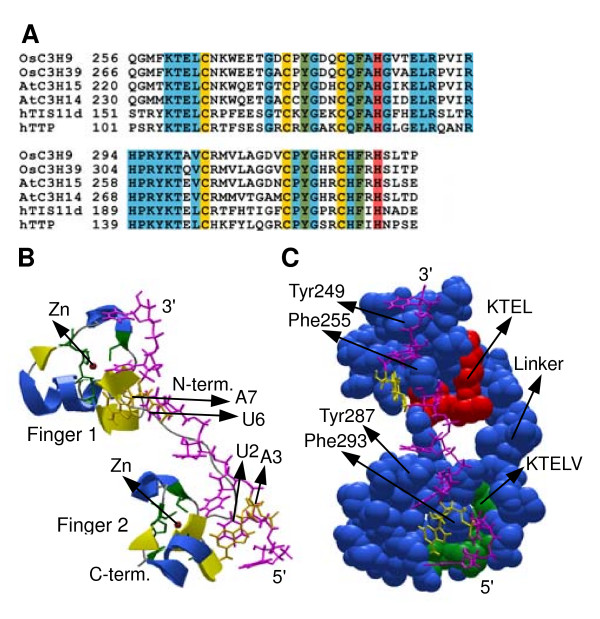
**Sequence alignments and the structure of AtC3H14 tandem domains in complex with the RNA**. A, Alignment of AtC3H14, AtC3H15, OsC3H9, OsC3H39, hTTP and hTIS11d. Conserved motifs are highlighted in light blue, gold and red (cysteines and histidines involved in zinc binding, respectively), and green (aromatic residues involved in base-stacking interactions with RNA). h, *Homo sapiens*. B, Structure of the RNA complex of AtC3H14. This proposed structure was modelled on the original nuclear magnetic resonance structure describe by Hudson et al. [69], using their pdb coordinates and the Swiss-Model program. The RNA oligonucleotide is shown in magenta (A3 and A7 are shown in yellow) with the 5' and 3' ends indicated. The protein backbone are shown to represent the structure, with alpha helices are coloured royal blue, beta sheets are coloured yellow, the zinc-coordinating ligands are colored green and the zinc residues are coloured red. C, Molecular contact surface of AtC3H14 structure showing surface topology. Blue denotes convex surfaces. The location of the motifs that form the U6 and U2 binding pockets and the aromatic residues which are essential for high-affinity binding are indicated.

Recently, the structure of the CCCH domain from the TIS11D in complex with RNA monomer of AU-rich element (ARE) have been determined [69]. Sequences alignment show that among 135 CCCH proteins in Arabidopsis and rice, AtC3H14, AtC3H15, OsC3H9 and OsC3H39 share high amino acid sequence identities with TIS11D (Figure 11A). Like the TIS11D, each of these four proteins contains two tandem (C-X_8_-C-X_5_-C-X_3_-H) CCCH zinc fingers and a linker of 18 residues between the two zinc finger motifs. In addition, many other residues are strictly conserved. To obtain insights of the structure of plant CCCH zinc finger, we have taken the coordinates of the TIS11D CCCH domain complex and modelled the structure of the interaction of the AtC3H14 (AT1G66810) CCCH domain peptide with the RNA nonamer (5'-UUAUUUAUU-3'), using the Swiss-Model programs [70]. It is apparent that the RNA-binding domain of the AtC3H14 is likely to be identical in structure to that of TIS11D. Compared with the structure of TIS11D, the characteristic KTEL(V) motif at the N-terminus of each zinc finger domain provides a critical part of the RNA-binding surface; each motif forms two walls of a deep pocket that accommodates the bases of U6 and U2 in fingers 1 and 2, respectively (Figure 11B). The model illustrates the aromatic stack formed from U2-Phe293-A3, U4-Tyr287-U5, U6-Phe255-A7 and U8-Tyr249-U9, which are essential for high-affinity binding. Mutation within these critical hydrophobic amino acids might make the domain abrogate RNA binding (Figure 11C). Further mutagenesis experiments would be required to establish the contributions of these residues to binding affinity.

In mammals, some proteins containing tandem CCCH domains, such as TTP and TIS11D, have been proved to bind to the class II ARE in the 3'-untranslated region (3'-UTR) of target mRNAs and promote their deadenylation and degradation [71]. AREs are sequence elements of 50–150 nt that are rich in adenosine and uridine bases. They are located in the 3'-UTRs of many but not all mRNAs that have a short half-life and have been identified by their capacity to provoke degradation of the host mRNA by a mechanism dependent on deadenylation. It has been estimated that 5–8% of human genes code for ARE-containing mRNAs, and the corresponding proteins perform a variety of functions implicated in numerous transient biological processes [72]. Henceforth, only two plant CCCH (HUA1 and AtCPSF30) proteins have been proved to have the RNA-binding ability by experimental evidence. Extraction of ARE-containing mRNAs in 3'-UTR of Arabidopsis genome was performed using Patmatch tools of TAIR. The 3'-UTRs were searched for the 13-bp pattern WWWU(AUUUA)UUUW. Totally, we identified 200 genes containing 258 ARE in their 3'-UTR mRNA (see Additional file 13). The potential relationship between the plant CCCH proteins and the AU-rich mRNA need been confirmed by experiment approaches in the future.

## Conclusion

In this study, we developed a new method employed regular expression of Perl to identify the gene family. By this method, 68 and 67 CCCH genes were identified in Arabidopsis and rice, respectively, and they formed 20 subfamilies that were supported by phylogeny, protein motifs and intron/exon structures. Compared with other gene families in rice and Arabidopsis, the CCCH gene family is one of the largest families in plants. Gene duplication analysis revealed that during the expansion of the CCCH gene family, many subfamilies and subgroups have evolved, resulting in a high level of functional divergence. Most of the subfamilies/subgroups are present both in Arabidopsis and rice, suggesting that the appearance of many of the genes in these species predates monocot/eudicot divergence. Members within a given subfamily/subgroup may have recent common evolutionary origins and may possess common zinc finger motifs that have related molecular functions. Available data supported the hypothesis that CCCH genes in plants perform a variety of functions in different tissues at multiple developmental stages. The subfamily IX of Arabidopsis with many interesting characters was investigated in details, in which members are shown to be involved in plant stress responses. We then proposed that the genes of subfamily IX possibly play roles as nucleocytoplasmic shuttle proteins involved in signal transduction events. Although the function of most plant CCCH genes is unknown, the phylogenetic and expression analyses provide a solid foundation for future functional studies in both Arabidopsis and rice. Overall, we have identified a novel zinc finger family and results presented here can serve as useful information for guiding future experimental work and understanding the structure-function relationship of the members of the CCCH gene family.

## Methods

### Identification of CCCH protein in Arabidopsis and rice

A preliminary search for CCCH proteins was performed using a program written by Perl. This program could search against the CCCH proteins with the regular expression in entire Arabidopsis and rice genome file which was downloaded from the FTP of TAIR [52] and TIGR [73]. The regular expression (C\w{6,14}C\w{4,5}C\w{3}H) was designed according to the previous studies. All of the proteins which matched the expression would be considered as candidate CCCH proteins. The obtained protein sequences were then examined for the CCCH motif using the hidden Markov model of SMART/Pfam tool. The proteins without CCCH motif were eliminated from the datasets. The new type motif which was not referred by the previous studies was used to modify the regular expression. The modified regular expression was used to retrieve for another round of searching, and new results were added to the original dataset.

Another approach of retrieving CCCH genes from database was employed. Multiple database searches were performed using the Basic Local Alignment Search Tool algorithms BLASTP and TBLASTN with various published CCCH proteins as query sequences and with the E-value cutoff set as 1e-005. For Arabidopsis, the databases searched included NCBI [51], DATF [74], MAtDB [75], TAIR [52], and TIGR [73] and for rice, the databases searched included NCBI [51], TIGR [76], Rice Genome Database-japonica of the Rice Genome Research Program [77] and The International Rice Genome Sequencing Project (IRSGP) [78]. The redundant sequences with different identification numbers and the same chromosome locus were removed from our data set.

The hits obtained from all the above methods were pooled together and another program written by Perl was performed to eliminate the redundant dataset. Some programs used in this research are listed in additional file (see Additional file 1, 2, 3, 4, 5 and 7).

### Sequence properties of Arabidopsis and rice CCCH genes

The amino acid sequences of the CCCH proteins were analyzed for physicochemical parameters (ProtParam) and predicted subcellular localization (SubLoc v1.0) on DBSubLoc [79]. MEME (Multiple Expectation Maximization for Motif Elicitation) was used to identify conserved motif structures of CCCH protein sequences.

### Alignment and phylogenetic analysis of CCCH sequences

Multiple alignments of amino acid sequences were performed using ClustalX and were manually corrected. For generating the phylogenetic tree, we used ClustalX (1.83) and the neighbor-joining algorithm. Bootstrap analysis with 1,000 replicates was used to evaluate the significance of the nodes. Representations of the calculated trees were constructed using TreeView. The phylogenetic trees of Figure 9 were constructed by neighbor-joining algorithms of MEGA3.1. Bootstrapping was performed 1000 times to obtain support values for each branch.

### The location of CCCH genes on chromosomes

To determine the location of CCCH genes on Arabidopsis chromosomes, Chromosome Map Tool at TAIR was used [50]. Gene duplications and their presence on duplicated chromosomal segments were investigated using "Paralogous in Arabidopsis" with the default parameters set to a minimum threshold for paired proteins per block above 7 [80]. For rice, all the sequenced contigs of *japonica *cv Nipponbare have been physically constructed as pseudomolecules by the IRGSP, representing the 12 rice chromosomes, and available in GenBank. Each of the rice CCCH genes was positioned on these rice chromosome pseudomolecules by the BLASTN search.

### Other plant species

To identify members of the moss CCCH protein family, multiple database searches were performed using the stand-alone BLAST tools available on NCBI. The EST database of moss was obtained on the *Physcomitrella *EST Project Web site [81], and then the file of results was parsed by a program written by Perl. For CCCH members of pine, the same method was used to search against the database got from NSF Genomics of Loblolly Pine Embryogenesis Project [56].

### Expression analysis of CCCH genes

We used MPSS and EST data to detect the expression patterns of CCCH genes. The locus name of CCCH genes were used to query the MPSS database containing the signature information of the CCCH genes. EST data came from UniGene of NCBI, TIGR and TAIR. We also searched the expression data of Arabidopsis CCCH genes in the database of Genevestigator [68].

### RT-PCR analysis

Plant tissues of Arabidopsis were harvested and ground in liquid nitrogen. For reverse transcriptase-mediated PCR analysis, total RNA was isolated with the RNeasy mini kit (Qiagen, USA) according to the manufacturer's instructions. The RNA preparation was then treated with DNase I. First strand synthesis of cDNA was performed by using oligo (dT) primer and M-MLV RT (Promega). PCR products were fractionated on 1% agarose gels containing ethidium bromide and photographed under UV light. These experiments were independently replicated at least three times under identical conditions. Details of primers are listed in the Additional file 14.

### Structure model

The model of complex of plant CCCH protein with the RNA nonamer was constructed using the Swiss-Model programs [70]. The final merged files was presented and annotated with Swiss Pdb Viewer 3.7 and Rastop 2.2.

## Authors' contributions

D.W. carried out all the analyses and interpreted the results. D.W. and Y.Y.L. jointly wrote the manuscript. Y.H.G. and C.C.Z contributed with the CCCH gene family background knowledge and edited the manuscript. All authors read and approved the final manuscript.

## Supplementary Material

Additional file 1 Figure S1. The program imports the BLAST result including the query sites and subject sites to the excel file. Click here for file

Additional file 2 Figure S2. The program reads dataset from "TAIR6_pep_20060907" (the file of Arabidopsis proteome) or "rice.pep" (the file of rice proteome) and input it into MySQL local database. Click here for file

Additional file 3 Figure S3. The program detects the putative CCCH proteins from Arabidopsis proteome. Click here for file

Additional file 4 Figure S4. The program sorts the CCCH motifs by their orders in CCCH protein sequences. Click here for file

Additional file 5 Figure S5. The program parses the database to search motifs that contain each other. Click here for file

Additional file 6 Table S1. Detailed information of CCCH gene family in Arabidopsis. Click here for file

Additional file 7 Figure S6. The program detects the putative CCCH proteins from rice proteome. Click here for file

Additional file 8 Table S2. Detailed information of CCCH gene family in rice. Click here for file

Additional file 9 *Figure S7. Phylogenetic trees of CCCH gene family*. A, Phylogenetic tree of rice CCCH gene family. The unrooted tree, constructed using ClustalX (1.83), summarizes the evolutionary relationship among the members of CCCH families in rice. The neighbor-joining tree was constructed using aligned full-length amino acid sequences. The tree shows the 8 major phylogenetic subfamilies (numbered I to VIII and marked with different alternating tones of a gray background to make subfamily identification easier) with high predictive value. Numbers on branches indicate the bootstrap values (1000 replicates) that support the adjacent node. The gene structure of rice are shown on the right side (boxes represent exons and lines represent introns). B, Joined phylogenetic tree of the rice and Arabidopsis CCCH gene families. The unrooted tree was inferred by the neighbor-joining method after the alignment of the full-length amino acid sequences of the 135 Arabidopsis and rice genes listed in Table 2 and Table 3 respectively. The tree shows the 20 phylogenetic subfamilies (numbered I to XX and marked with different alternating tones of a gray background to make subfamily identification easier) with high predictive value. Numbers on branches indicate the bootstrap values (1000 replicates) that support the adjacent node. Click here for file

Additional file 10 Figure S8. Chromosomal distribution for rice CCCH genes. Click here for file

Additional file 11 Figure S9. The program detects the putative NES sequences form CCCH proteins. Click here for file

Additional file 12 Table S3: The putative NES sequences in CCCH proteins detecting by regular expression consensus [LV]-x(2,3)-[LIVFM]-x(2,3)-L-x-[LIMTKD] Click here for file

Additional file 13 *Table S4. The gene locus containing *WWWT(ATTTA)TTTW *pattern in 3'-UTR of Arabidopsis genome*. Click here for file

Additional file 14 Table S5: Primers for RT-PCRs. Click here for file
