# Supplementary material for: Genome-wide analysis of CCCH zinc finger family in Arabidopsis and rice
Source: BMC Genomics. 2008 Jan 27;9:44. doi: 10.1186/1471-2164-9-44 (PMC2267713; doi:10.1186/1471-2164-9-44)
Supplement: Additional file 1 — Figure S1. The program imports the BLAST result including the query sites and subject sites to the excel file. [file 1471-2164-9-44-S1.pdf]

Supplemental Figure S1.

```perl
#!/usr/bin/perl
#!The program imports the BLAST result including the Query sites and Sbject sites to the excel file.
use Getopt::Std;
getopts "o:i:e:a:l:s:z:d:m:";

print "*************\n*$vision*\n*************\n";
if ((!defined $opt_i)|| (!defined $opt_o) ) {
    die "**********************************************************************
    Usage:transferall.pl -i filename -o outfile.
      -h : help and usage.
      -v : vision.
      -e : expect value(default 10)
      -a : identity% (default 10)
      -l : alignment length (default 0)
      -s : score (default 0)
      -z : the block number of every query  (default 10000)
      -d : the block number of every sbjct  (default 10000)
      -m : detailed description of sbjct y/n (defaule n)
**********************************************************************\n";
}
if($opt_i eq $opt_o) { die"infile = outfile?"; }
$Expect = (defined $opt_e) ? $opt_e : 10;
$Length = (defined $opt_l) ? $opt_l : 0;
$Identity = (defined $opt_a) ? $opt_a : 10;
$Score = (defined $opt_s) ? $opt_s : 0;
$Query_num = (defined $opt_z) ? $opt_z : 10000;
$Sbjct_num = (defined $opt_d) ? $opt_d : 10000;
$Sbjct_description = (defined $opt_m) ? $opt_m : "n";

open(Ofile,">$opt_o");print "Running.....\n";
open (F,$opt_i) || die"can't open $opt_i:$!\n";
$i=$q=$s=$a=0;
if($Sbjct_description eq "y")
{
    printf Ofile "Query-name\tLetter\tQueryX\tQueryY\tSbjctX\tSbjctY\tLength\tScore\tE-value\tOverlap/total\tIdentity\tSbject-Name\tQuery_sequence\tSbjct_description\n" ;
}
elsif($Sbjct_description eq "n")
{
    printf Ofile "Query-name\tLetter\tQueryX\tQueryY\tSbjctX\tSbjctY\tLength\tScore\tE-value\tOverlap/total\tIdentity\tSbject-Name\n" ;
}
else { die"\$Sbjct_description(-m) should be y/n.\n"; }

while (<F>)
{
   if (/Query= \s?(\S+)/)
   {
        if($i==1)
        {
            #print Ofile "$query^$letter^Query:$qbegin----$qend^Sbject:$sbegin----$send^$name^$annotation^$length^$score^$expect^$identity^$over\n";
            if($score >$Score && $expect<=$Expect && $over>=$Identity && $identity2 >= $Length && $query_num <= $Query_num && $sbjct_num <= $Sbjct_num)
            {
                $ovalap_total = "$identity1/$identity2";
                if($Sbjct_description eq "y")
                {
                    print Ofile "$query\t$letter\t$qbegin\t$qend\t$sbegin\t$send\t$length\t$score\t$expect\t$ovalap_total\t$over\t$name\t$query_seq\t$annotation\n" ;
                }
                elsif($Sbjct_description eq "n")
                {
                    print Ofile "$query\t$letter\t$qbegin\t$qend\t$sbegin\t$send\t$length\t$score\t$expect\t$ovalap_total\t$over\t$name\n" ;
                }
            }
            $i=$q=$s=0;
            $query=$letter=$qbegin=$qend=$sbegin=$send=$name=$annotation=$length=$score=$expect=$identity=$over=0;
        }

        $query=$1;
        $query_num = 0;
    }
    elsif (/\((\S+)\s+letters\)/)
    {
        $letter=$1;$letter=~s/,//;
    }
    elsif (/^>(\S*)\s*(.*)/)
    {
        if($i==1)
        {
            if($score >$Score && $expect<=$Expect && $over>=$Identity && $identity2 >= $Length && $query_num <= $Query_num && $sbjct_num <= $Sbjct_num)
            {
                $ovalap_total = "$identity1/$identity2";
                if($Sbjct_description eq "y")
                {
                    print Ofile "$query\t$letter\t$qbegin\t$qend\t$sbegin\t$send\t$length\t$score\t$expect\t$ovalap_total\t$over\t$name\t$query_seq\t$annotation\n" ;
                }
                elsif($Sbjct_description eq "n")
                {
                    print Ofile "$query\t$letter\t$qbegin\t$qend\t$sbegin\t$send\t$length\t$score\t$expect\t$ovalap_total\t$over\t$name\n" ;
                }
            }
            $i=$q=$s=0;
            $qbegin=$qend=$sbegin=$send=$name=$annotation=$length=$score=$expect=$identity=$over=0;
        }
        $name=$1;
        $annotation=$2;
        $a=1;
        $sbjct_num = 0;
    }
   elsif(/Length\s?=\s?(\d+)/){
        $length=$1;
        $a=0;
    }
    elsif ($a==1)
    {
        chomp;
        $annotation.=$_;
        $annotation=~s/\s+/ /g;
    } #This sentence could get the very long annotation that is longer than one line;
    elsif (/Score = (.+) bits.+Expect\S* =\s+(\S+)\s*/)
    {
        if($i==1)
```

Supplemental Figure S1.

```
        {
            if($score >$Score && $expect<=$Expect && $over>=$Identity && $identity2 >= $Length && $query_num <= $Query_num && $sbjct_num <= $Sbjct_num)
            {
                $ovalap_total = "$identity1/$identity2";
                if($Sbjct_description eq "y")
                {
                    print Ofile "$query\t$letter\t$qbegin\t$qend\t$sbegin\t$send\t$length\t$score\t$expect\t$ovalap_total\t$over\t$name\t$query_seq\t$annotation\n" ;
                }
                elsif($Sbjct_description eq "n")
                {
                    print Ofile "$query\t$letter\t$qbegin\t$qend\t$sbegin\t$send\t$length\t$score\t$expect\t$ovalap_total\t$over\t$name\n" ;
                }
            }
            $i=$q=$s=0;
            $qbegin=$qend=$sbegin=$send=$score=$expect=$identity=$over=0;
        }
        $query_num++;
        $sbjct_num++;
        $score=$1;$expect=$2;$expect=~s/^e/1e/;
    }
    elsif (/Identities = (\d+)\/(\d+)\s+\((.{0,4})%\)/)
    {
        $identity1=$1;
        $identity2=$2;
        $over=$3;
    }
   elsif (/Query((\:\s+)|\s+)(\d+)\s*(\S+)\s+(\d+)/){
        if($q==0)
        {
            $qbegin=$3;
            $query_seq = $4;
        }
        else { $query_seq .= $4; }
        $qend=$5;
        $q=1;
    }
    elsif (/Sbjct((\:\s+)|\s+)(\d+)\s*(\S+)\s+(\d+)/){
        if($s==0)
        {
            $sbegin=$3;
        }
        $send=$4;
        $s=$i=1;
    }
}
if(($score >$Score && $expect<=$Expect && $over>=$Identity && $identity2 >= $Length && $query_num <= $Query_num && $sbjct_num <= $Sbjct_num)&&($i==1))
{
    $ovalap_total = "$identity1/$identity2";
    if($Sbjct_description eq "y")
    {
        print Ofile "$query\t$letter\t$qbegin\t$qend\t$sbegin\t$send\t$length\t$score\t$expect\t$ovalap_total\t$over\t$name\t$query_seq\t$annotation\n" ;
    }
    elsif($Sbjct_description eq "n")
    {
        print Ofile "$query\t$letter\t$qbegin\t$qend\t$sbegin\t$send\t$length\t$score\t$expect\t$ovalap_total\t$over\t$name\n" ;
    }
}
close(F);
close(Ofile);
```
