# Supplementary material for: Genome-wide analysis of CCCH zinc finger family in Arabidopsis and rice
Source: BMC Genomics. 2008 Jan 27;9:44. doi: 10.1186/1471-2164-9-44 (PMC2267713; doi:10.1186/1471-2164-9-44)
Supplement: Additional file 2 — Figure S2. The program reads dataset from "TAIR6_pep_20060907" (the file of Arabidopsis proteome) or "rice.pep" (the file of rice proteome) and input it into MySQL local database. [file 1471-2164-9-44-S2.pdf]

Supplemental Figure S2.

```perl
#!/usr/bin/perl
use DBI;
use DBD::mysql;
#!The program reads dataset from "TAIR6_pep_20060907" (the file of Arabidopsis proteome) or "rice.pep" (the file of rice proteome) and input it into MySQL local database.
my $dbh=DBI->connect("DBI:mysql:ccch","root","");
open(F,"TAIR6_pep_20060907");
my $pep;
my $i=0;
my @acc_array;
while(my $line=<F>)
{

   if($line=~m/>/)
    {
       $name=substr($line,1,11);#!arabidopsis
       #!$name=substr($line,1,16);#!rice
       $i++;
       push(@acc_array,$name);
       print $i.$name."\n";
    }
    else
    {
      chomp($line);
    $$name=$$name.$line

    }

   }

close F;
 foreach $accession(@acc_array)
 {
#!input the whole genome into table (arabidopsis)
$newaccession=substr($accession,0,9);
    $insert_sql="insert into arabidopsis (accession,daccession,pep) values ('$accession','$newaccession','$$accession')" ;
     $dbh->do($insert_sql);
 }
```
