# Supplementary material for: Genome-wide analysis of CCCH zinc finger family in Arabidopsis and rice
Source: BMC Genomics. 2008 Jan 27;9:44. doi: 10.1186/1471-2164-9-44 (PMC2267713; doi:10.1186/1471-2164-9-44)
Supplement: Additional file 4 — Figure S4. The program sorts the CCCH motifs by their orders in CCCH protein sequences. [file 1471-2164-9-44-S4.pdf]

Supplentment Figure S4.

```
#!/usr/bin/perl
#!/usr/bin/perl
#!The program sorts the CCCH motifs by their orders in the protein sequences.
use DBI;
use DBD::mysql;
my $dbh=DBI->connect("DBI:mysql:ccch","root","");
my $string="select * from final1";
my $sth=$dbh->prepare($string);
$sth->execute();
my $i=0;
#!open(WANG,">>remotif.txt");
LABEL:while(my @row=$sth->fetchrow_array())
{
    @idmotifarray=();
    @idarray=();
    my $arraynum=0;
    $accession=@row[1];
    $motif=@row[4];
    $num=@row[3];
    $pep=@row[5];
    $style=@row[6];
    @stylearray = split(/\|/, $style);
    @array = split(/\|/, $motif);
    $arraynum=((scalar(@array))-1);
    for ($j=1;$j<=$arraynum;$j++) {
        @array2=split(/:/, @array[$j]);;
        $id=@array2[0];
        $idmotif=@array2[1];
        push(@idarray,$id);
        push(@idmotifarray,$idmotif);
    }
   print "@stylearray"."\n";print "@idmotifarray"."\n";
   for($n=0;$n<$arraynum;$n++){
       for ($k=$n+1;$k<$arraynum;$k++) {
           $pos1=index($pep,@idmotifarray[$n],0);
           $pos2=index($pep,@idmotifarray[$k],0);
           if($pos2<$pos1)
           {
               $temp=@idmotifarray[$n];
               @idmotifarray[$n]=@idmotifarray[$k];
               @idmotifarray[$k]=$temp;
               $temp2=@stylearray[$n+1];
               @stylearray[$n+1]=@stylearray[$k+1];
               @stylearray[$k+1]=$temp2;
           }
         }
      }print "@stylearray"."\n";print "@idmotifarray"."\n";
    my $newstyle=$newmotif="|";
    my $deal=0;
    foreach(@stylearray)
    {      if($deal!=0)
        {
           $newstyle=$newstyle.$_."|";
        }
        $deal++;
    } print $newstyle;
    print "\n";
    $order=0;
    foreach(@idmotifarray)
    {      $order++;
           $newmotif=$newmotif."ZF".$order.":".$_."|";

    } print $newmotif;

           my $upstring="update final1 set motif='$newmotif',style='$newstyle' where accession='$accession'" ;
  $dbh->do($upstring);
}
```
