# Supplementary material for: Genome-wide analysis of CCCH zinc finger family in Arabidopsis and rice
Source: BMC Genomics. 2008 Jan 27;9:44. doi: 10.1186/1471-2164-9-44 (PMC2267713; doi:10.1186/1471-2164-9-44)
Supplement: Additional file 5 — Figure S5. The program parses the database to search motifs that contain each other. [file 1471-2164-9-44-S5.pdf]

Supplentment Figure S5.

```perl
#!/usr/bin/perl
#!/usr/bin/perl
#!The program parses the database to search motifs that contain each other.
use DBI;
use DBD::mysql;
my $dbh=DBI->connect("DBI:mysql:ccch","root","");
my $string="select * from final1";
my $sth=$dbh->prepare($string);
$sth->execute();
my $i=0;
open(WANG,">>remotif.txt");
LABEL:while(my @row=$sth->fetchrow_array())
{
    @idmotifarray=();
    @idarray=();
    my $arraynum=0;
    $accession=@row[1];
    $motif=@row[4];
    $num=@row[3];
    $style=@row[6];
    @stylearray = split(/\|/, $style);
    #!print @stylearray[6];
    @array = split(/\|/, $motif);
    $arraynum=((scalar(@array))-1);
    for ($j=1;$j<=$arraynum;$j++) {
        @array2=split(/:/, @array[$j]);;
        $id=@array2[0];
        $idmotif=@array2[1];
        push(@idarray,$id);
        push(@idmotifarray,$idmotif);

    }
   for($n=0;$n<$arraynum;$n++){
       for ($k=$n+1;$k<$arraynum;$k++) {
           if((@idmotifarray[$k]=~m/(@idmotifarray[$n])/)||(@idmotifarray[$n]=~m/(@idmotifarray[$k])/))
           {
               $i++;
               print WANG $i."accession:".$accession."\n";
               print WANG "num:".$num."\n";
               print WANG "motif:".$motif."\n";
               print WANG "style:".$style."\n";
               print WANG @stylearray[$n+1].":".@idmotifarray[$n]."---".@stylearray[$k+1].":".@idmotifarray[$k];
               print WANG "\n"."++++++++++++++++++++++"."\n";
               }
         }
      }
}

print WANG $i;
```
