# Supplementary material for: Genome-wide analysis of CCCH zinc finger family in Arabidopsis and rice
Source: BMC Genomics. 2008 Jan 27;9:44. doi: 10.1186/1471-2164-9-44 (PMC2267713; doi:10.1186/1471-2164-9-44)
Supplement: Additional file 7 — Figure S6. The program detects the putative CCCH proteins from rice proteome. [file 1471-2164-9-44-S7.pdf]

Supplentment Figure S6.

```
#!/usr/bin/perl
use DBI;
use DBD::mysql;
#!The program detects the putative CCCH proteins from rice proteome.
my $dbh=DBI->connect("DBI:mysql:rice","root","");
my $string="select * from rice";
my $sth=$dbh->prepare($string);
$sth->execute();
my $i=0;#!
my $wholzincnum=0;
LABEL: while(my @row=$sth->fetchrow_array())
{
   my $znum=0;
   my $pep=@row[1];
   my $wpep=$pep;
   my $accession=@row[0];
   my $motif="|";
 for $space1 (4..14)
  {

    for $space2 (4..6)
        {
           my $motiflength=$space1+$space2+3+3;
           my $name="CCCH".$space1.$space2."3";
           if(!$$name)
           {
              $$name=0;
           }
           if($wpep=~m/(C\w{$space1}C\w{$space2}C\w{3}H)/)
          {
                     $$name++;
                     $wholzincnum++;
                     $znum++;
                     $i++;
                     print $i."------".$accession."ccch".$space1.$space2."3";
                     print $&."\n";
                     $motif=$motif."ZF".$znum.":".$&."|";
          LOOP: while((length($pep)>=$motiflength)&&($pep=~m/(C\w{$space1}C\w{$space2}C\w{3}H)/))
                     {
                       $findex=index($pep,$&,0);
                       $pep=substr($pep,($findex+$motiflength+1));

                        if($pep=~m/(C\w{$space1}C\w{$space2}C\w{3}H)/)
                         {
                          $wholzincnum++;
                          $znum++;
                         print $&."\n";
                          $motif=$motif."ZF".$znum.":".$&."|";
                          next LOOP;
                         }


                     }

  $pep=$wpep;

          }
        }

   }
              if($wpep=~m/(C\w{4,14}C\w{4,6}C\w{3}H)/)
          {
               print "\n".$motif."\n";
               $newaccession=substr($accession,0,9);
               $insert_sql="insert into atccch (accession,daccession,num,motif,pep) values ('$accession','$newaccession','$znum','$motif','$wpep')";
               $dbh->do($insert_sql);
          }
}


print "+++++++++++++++++++++".$wholzincnum."+++++++++++++++++++++";
print "\n";
 for $space1 (4..14)
  {

    for $space2 (4..6)
        {

           my $name="CCCH".$space1.$space2."3";
           print "CCCH".$space1.$space2."3:".$$name;
           print "\n";
        }
  }
```
