# Supplementary figures and images for: Genome-wide analysis of CCCH zinc finger family in Arabidopsis and rice
Source: BMC Genomics. 2008 Jan 27;9:44. doi: 10.1186/1471-2164-9-44 (PMC2267713; doi:10.1186/1471-2164-9-44)

**Figure S7.**


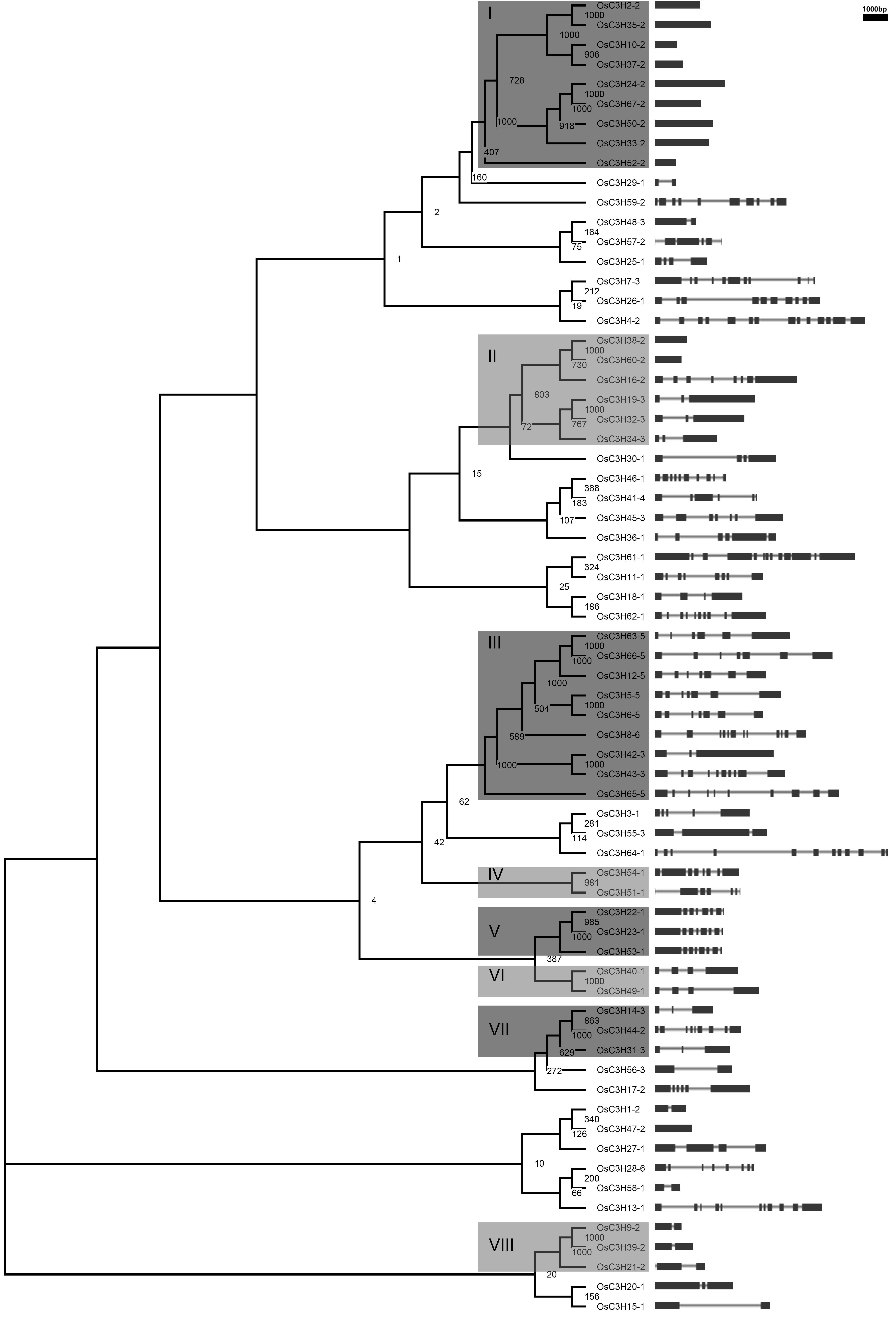


**A**


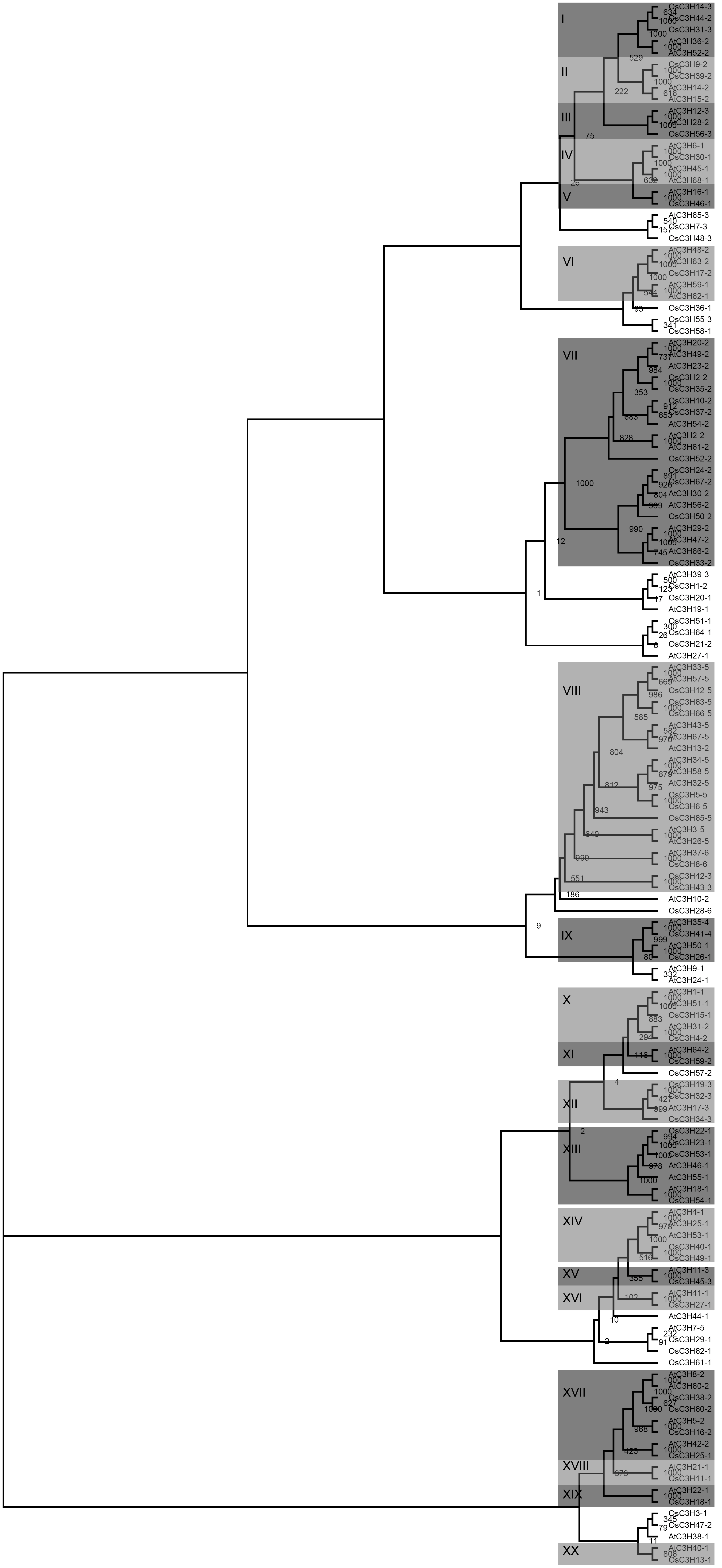


**B**

Supplement: Additional file 9 — Figure S7. Phylogenetic trees of CCCH gene family. A, Phylogenetic tree of rice CCCH gene family. The unrooted tree, constructed using ClustalX (1.83), summarizes the evolutionary relationship among the members of CCCH families in rice. The neighbor-joining tree was constructed using aligned full-length amino acid sequences. The tree shows the 8 major phylogenetic subfamilies (numbered I to VIII and marked with different alternating tones of a gray background to make subfamily identification easier) with high predictive value. Numbers on branches indicate the bootstrap values (1000 replicates) that support the adjacent node. The gene structure of rice are shown on the right side (boxes represent exons and lines represent introns). B, Joined phylogenetic tree of the rice and Arabidopsis CCCH gene families. The unrooted tree was inferred by the neighbor-joining method after the alignment of the full-length amino acid sequences of the 135 Arabidopsis and rice genes listed in Table 2 and Table 3 respectively. The tree shows the 20 phylogenetic subfamilies (numbered I to XX and marked with different alternating tones of a gray background to make subfamily identification easier) with high predictive value. Numbers on branches indicate the bootstrap values (1000 replicates) that support the adjacent node. [file 1471-2164-9-44-S9.doc]

**
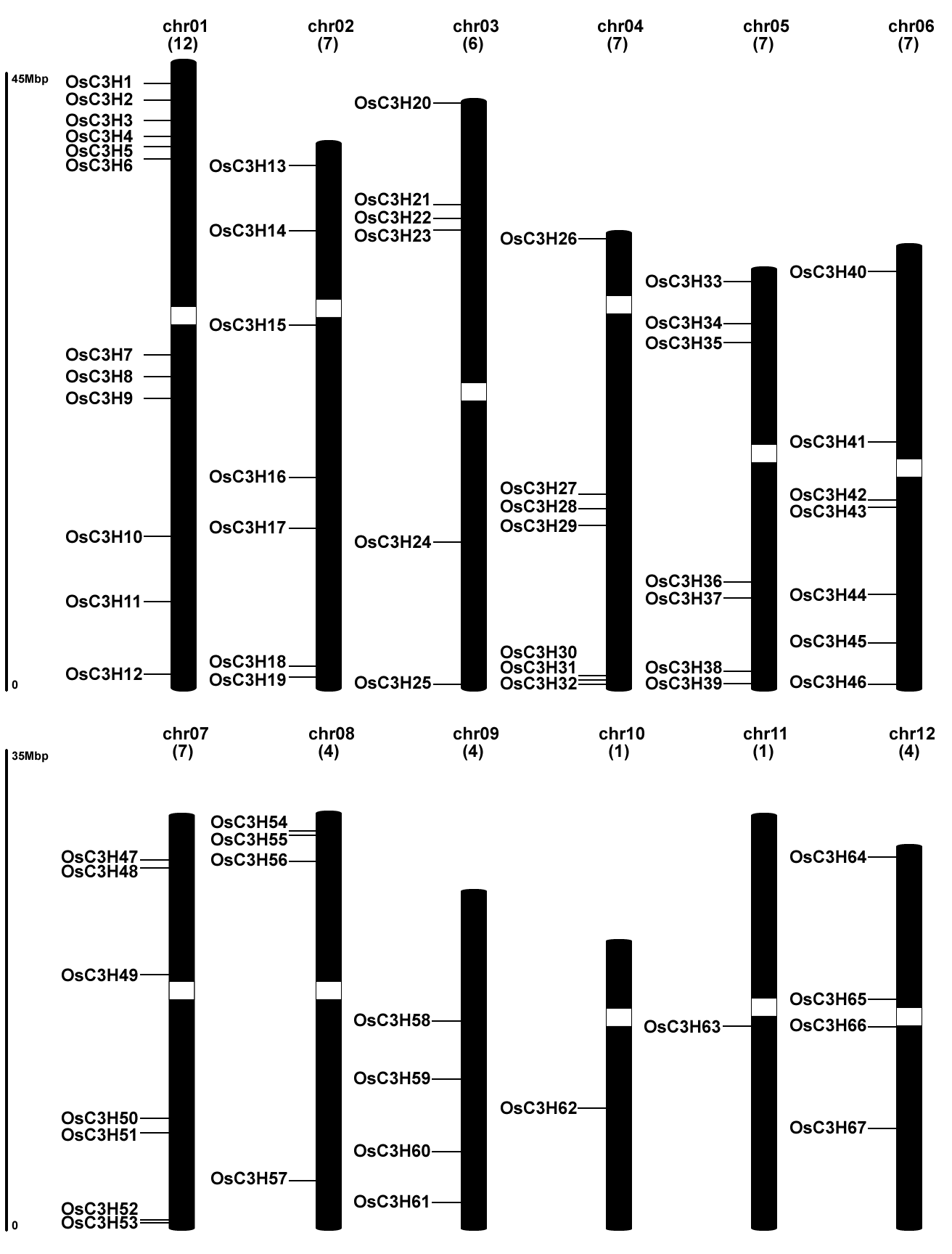
Figure S8.**

Supplement: Additional file 10 — Figure S8. Chromosomal distribution for rice CCCH genes. [file 1471-2164-9-44-S10.doc]
