# Supplementary material for: Genome-wide analysis of CCCH zinc finger family in Arabidopsis and rice
Source: BMC Genomics. 2008 Jan 27;9:44. doi: 10.1186/1471-2164-9-44 (PMC2267713; doi:10.1186/1471-2164-9-44)
Supplement: Additional file 11 — Figure S9. The program detects the putative NES sequences form CCCH proteins. [file 1471-2164-9-44-S11.pdf]

Supplentment Figure S9.

```perl
#!/usr/bin/perl
use DBI;
use DBD::mysql;
#!The program detects the putative NES sequences form CCCH proteins.
my $dbh=DBI->connect("DBI:mysql:ccch","root","");
my $string="select * from final3";
my $sth=$dbh->prepare($string);
$sth->execute();
my $i=0;#!
my $k=0;
LABEL: while(my @row=$sth->fetchrow_array())
{
  my $pep=@row[5];
  my $wpep=$pep;
  my $en=@row[0];
  my $daccession=@row[2];
  my $en=@row[0];
 for $space1 (2..3)
  {

    for $space2 (2..3)
        {
           my $motiflength=$space1+$space2+1+3;
           my $name="NES".$space1.$space2."3";
           if(!$$name)
           {
              $$name=0;
           }
           if($wpep=~m/((L|V)\w{$space1}(L|I|V|F|M)\w{$space2}L\w{1}(M|T|K|D|I|L))/)
          {
                     $$name++;
                     $i++;
                     print $i."------".$en."NES".$space1.$space2."3";
                     print $&."\n";
          LOOP: while((length($pep)>=$motiflength)&&($pep=~m/((L|V)\w{$space1}(L|I|V|F|M)\w{$space2}L\w{1}(M|T|K|D|I|L))/))
                     {
                       $findex=index($pep,$&,0);
                       $pep=substr($pep,($findex+$motiflength+1));
                        if($pep=~m/((L|V)\w{$space1}(L|I|V|F|M)\w{$space2}L\w{1}(M|T|K|D|I|L))/)
                         {
                         print $&."\n";
                          next LOOP;
                         }


                     }

  $pep=$wpep;

          }
        }

   }

}
print "\n";
 for $space1 (2..3)
  {

    for $space2 (2..3)
        {

           my $name="NES".$space1.$space2."3";
           print "NES".$space1.$space2."3:".$$name;
           print "\n";
        }
  }
```
