# Supplementary material for: Genome-wide analysis of CCCH zinc finger family in Arabidopsis and rice
Source: BMC Genomics. 2008 Jan 27;9:44. doi: 10.1186/1471-2164-9-44 (PMC2267713; doi:10.1186/1471-2164-9-44)
Supplement: Additional file 12 — Table S3: The putative NES sequences in CCCH proteins detecting by regular expression consensus [LV]-x(2,3)-[LIVFM]-x(2,3)-L-x-[LIMTKD] [file 1471-2164-9-44-S12.doc]

***Table S3:*** *The putative NES sequences in Arabidopsis CCCH proteins detecting by regular expression consensus [LV]-x(2,3)-[LIVFM]-x(2,3)-L-x-[LIMTKD].*

The sequences are searched by Perl program which is available in Additional file 7.

| **NO.** | **Gene Name** | **Putative NES Sequences** | **No.** | **Gene Name** | **Putative NES Sequences** |
| --- | --- | --- | --- | --- | --- |
| 1 | AtC3H1 | VGPCIKRALEK | 49 | AtC3H40 | LQKLGNQLST |
| 2 | AtC3H2 | LGWVNDLLT | 50 | AtC3H40 | VEMDVKMLTD |
| 3 | AtC3H3 | VMFNVIGLPM | 51 | AtC3H40 | LTSRIQELET |
| 4 | AtC3H4 | VCQVCLLDL | 52 | AtC3H41 | VPILKPLST |
| 5 | AtC3H5 | LNDSISDLST | 53 | AtC3H41 | LERLKETLRK |
| 6 | AtC3H7 | LISIYNLIT | 54 | AtC3H41 | VPPLPTGLAD |
| 7 | AtC3H11 | LQKIQQLTT | 55 | AtC3H41 | VDENVAPLDD |
| 8 | AtC3H11 | VLQKIQQLTT | 56 | AtC3H41 | LRPPLNAPLNM |
| 9 | AtC3H11 | VGEQLASLLYL | 57 | AtC3H41 | VVPPLPTGLAD |
| 10 | AtC3H14 | LNRLQSLMT | 58 | AtC3H42 | LTQVKNLQK |
| 11 | AtC3H15 | LRMLSNLSI | 59 | AtC3H42 | VDNLNGALVL |
| 12 | AtC3H17 | LESDLRYRLAK | 60 | AtC3H44 | LFKVYWLCL |
| 13 | AtC3H18 | LSYLLDYLNT | 61 | AtC3H44 | VGHFEMLKL |
| 14 | AtC3H18 | LEGEIIELLK | 62 | AtC3H44 | VISIDQLSD |
| 15 | AtC3H18 | LEGEIIELLKL | 63 | AtC3H44 | LTVVDILKT |
| 16 | AtC3H19 | LRECVEKLQL | 64 | AtC3H44 | VMLEILNLDK |
| 17 | AtC3H20 | VASLRNLQL | 65 | AtC3H44 | LRRKFLESLLD |
| 18 | AtC3H20 | VGWVSDLVM | 66 | AtC3H44 | LRQSIKCGLNK |
| 19 | AtC3H20 | VVASLRNLQL | 67 | AtC3H45 | LTGLAPDLSL |
| 20 | AtC3H20 | LGPGFRSLPT | 68 | AtC3H46 | LPERLEDSLPD |
| 21 | AtC3H20 | LRAQLFEKLSK | 69 | AtC3H47 | VSWVNSLVK |
| 22 | AtC3H21 | LDELEASLSK | 70 | AtC3H48 | LVGMPNLVK |
| 23 | AtC3H23 | LGFFSGLAT | 71 | AtC3H48 | LLVGMPNLVK |
| 24 | AtC3H24 | VSVVDLSLSK | 72 | AtC3H49 | VGWVSDLLM |
| 25 | AtC3H24 | VATEFEELAK | 73 | AtC3H49 | LGPGFQSLPT |
| 26 | AtC3H24 | LHPAVIKALRT | 74 | AtC3H49 | LREKMREKLHK |
| 27 | AtC3H25 | VCQVCLLDL | 75 | AtC3H50 | VSRVVELID |
| 28 | AtC3H26 | LFNVLGLPM | 76 | AtC3H50 | LIDFRDKLYL |
| 29 | AtC3H26 | VLFNVLGLPM | 77 | AtC3H53 | VCQVCLLDL |
| 30 | AtC3H29 | VEIIKILLD | 78 | AtC3H55 | LNDCLSFLDD |
| 31 | AtC3H29 | VSWVNSLVK | 79 | AtC3H56 | VEGFRRQLSD |
| 32 | AtC3H30 | VSWVQSLVK | 80 | AtC3H56 | LDAWLEQLHL |
| 33 | AtC3H30 | VIKLIVSLTD | 81 | AtC3H56 | LRTILEEILKK |
| 34 | AtC3H31 | LLSIQALRK | 82 | AtC3H61 | LGWVNDLLT |
| 35 | AtC3H31 | LVCVKQFLMK | 83 | AtC3H61 | LISSLDSLSL |
| 36 | AtC3H31 | LQNLIHDLIL | 84 | AtC3H63 | LVGMPNLVK |
| 37 | AtC3H31 | LGVLMDTLPL | 85 | AtC3H63 | LQCIQTLTD |
| 38 | AtC3H31 | LENLKQLLSK | 86 | AtC3H63 | LLVGMPNLVK |
| 39 | AtC3H31 | LYKIRVVLTM | 87 | AtC3H64 | LGLFYVELPD |
| 40 | AtC3H31 | LEANMAPILCT | 88 | AtC3H64 | VGESFYCALPK |
| 41 | AtC3H31 | VLVCVKQFLMK | 89 | AtC3H66 | LEIVTLLLK |
| 42 | AtC3H31 | VDDALRMLLSI | 90 | AtC3H66 | VSWVEPLVK |
| 43 | AtC3H31 | LLGVLMDTLPL | 91 | AtC3H66 | LSHLKFSLLL |
| 44 | AtC3H32 | LQESMWRLGL | 92 | AtC3H66 | VDYIISTGLVD |
| 45 | AtC3H34 | VEAPMWRLGL | 93 | AtC3H67 | VPELNFLGL |
| 46 | AtC3H35 | LSEFLGGLQI | 94 | AtC3H67 | LNHVSEELSD |
| 47 | AtC3H38 | VEVVKELLK | 95 | AtC3H68 | LCQVKLFLSD |
| 48 | AtC3H38 | LVEVVKELLK | 96 | AtC3H68 | VSGLGPELSL |
