# Supplementary material for: Genome-wide analysis of CCCH zinc finger family in Arabidopsis and rice
Source: BMC Genomics. 2008 Jan 27;9:44. doi: 10.1186/1471-2164-9-44 (PMC2267713; doi:10.1186/1471-2164-9-44)
Supplement: Additional file 14 — Table S5: Primers for RT-PCRs. [file 1471-2164-9-44-S14.doc]

***Table S5:*** *Primers for RT-PCRs*

| **Gene Name** | **Gene Identifier** | **Forward and Reverse Primer Sequences** |
| --- | --- | --- |
| AtC3H20 | AT2G19810 | CAACTACACCGACCCGACCT  and AGAGACCCACCCGACATCAG |
| AtC3H 49 | AT4G29190 | TCTCTGCCTACAACACCGACC  and ACTCAACGACACGCTCCATTACG |
| AtC3H 23 | AT2G25900 | CTTCGCTTCTCTCTTCCGTTACCT  and ATGAGTCGGTGAGTGGTGATGA |
| AtC3H 2 | AT1G03790 | CAACAACAACCAAGTAGCCCT  and TCACCACAAGAACCATCCTCA |
| AtC3H 61 | AT5G44260 | ATCCTTACGCTGGAGACCATTTCCG  and TGACGGCTAAACTCAGGGCATACT |
| AtC3H 54 | AT5G07500 | ACGGTGTCCTCGTACTCGTAGCCA  and GTGTCGTAACCCTCGTCGTCCATT |
| AtC3H 30 | AT2G41900 | TGTGCTCGGCGGGTTTGTTT  and ACCTTAGCCTGCTTGACTGTAGATTGC |
| AtC3H 56 | AT5G12850 | CTATCGCCTTCCCACAAGTCCG  and GGGAGCTTAGGCTGCGTGAATGTA |
| AtC3H 66 | AT5G58620 | TCTGCTGAAGGGCTCTGCGAATC  and CAACTATAATGGTACTTCCTCGGATCACG |
| AtC3H 29 | AT2G40140 | GATATGTGCGGTGCAAAGAGC  and GAGCTCTTATGCCACAATCTGCTGCTCA |
| AtC3H 47 | AT3G55980 | AGATGAAACGGGTTGTGCAAGGA  and AGCCGGTGGAGTAAGGGTATTGAC |
